# RNA-seq analysis reveals changes in mRNA expression during development in *Daphnia mitsukuri*

**DOI:** 10.1186/s12864-024-10210-8

**Published:** 2024-03-21

**Authors:** Xiuping Zhang, Wenwu Yang, David Blair, Wei Hu, Mingbo Yin

**Affiliations:** 1 MOE Key Laboratory for Biodiversity Science and Ecological Engineering, School of Life Science, Fudan University, Songhu Road 2005, Shanghai, China; 2 College of Marine and Environmental Sciences, James Cook University, Townsville Qld, 4811 Australia

**Keywords:** Development, Temporal, Transcriptomics, *Daphnia*, Embryo, Genes

## Abstract

Temporal transcriptional variation is a major contributor to functional evolution and the developmental process. Parthenogenetic water fleas of the genus *Daphnia* (Cladocera) provide an ideal model to characterize gene expression patterns across distinct developmental stages. Herein, we report RNA-seq data for female *Daphnia mitsukuri* at three developmental stages: the embryo, juvenile (three timepoints) and adult. Comparisons of gene expression patterns among these three developmental stages and weighted gene co-expression network analysis based on expression data across developmental stages identified sets of genes underpinning each of the developmental stages of *D. mitsukuri*. Specifically, highly expressed genes (HEGs) at the embryonic developmental stage were associated with cell proliferation, ensuring the necessary foundation for subsequent development; HEGs at the juvenile stages were associated with chemosensory perception, visual perception and neurotransmission, allowing individuals to enhance detection of potential environmental risks; HEGs at the adult stage were associated with antioxidative defensive systems, enabling adults to mount an efficient response to perceived environmental risks. Additionally, we found a significant overlap between expanded gene families of *Daphnia* species and HEGs at the juvenile stages, and these genes were associated with visual perception and neurotransmission. Our work provides a resource of developmental transcriptomes, and comparative analyses that characterize gene expression dynamics throughout development of *Daphnia*.

**Supplementary Information:**

The online version contains supplementary material available at 10.1186/s12864-024-10210-8.

## Introduction

The developmental processes of differentiation and growth exhibited by an organism reflect past evolutionary constraints and also influence future evolutionary options for the species [1, 2]. With the advance of high-throughput sequencing technology, it is now possible to obtain comprehensive transcriptomic information from any developmental stage of an organism. This will provide deeper understanding of the regulatory processes governing development [1]. A high-resolution analysis of gene expression during development has been undertaken, for example in *Drosophila melanogaster* [3, 4], *Caenorhabditis elegans* [5] and *Aedes aegypti* [6], and the functional transcripts associated with distinct developmental stages were identified. Graveley et al. [4] used RNA-Seq to explore transcriptomes in 27 distinct developmental stages of *D. melanogaster* and identified over 1,500 genes with pronounced expression changes during the larval stage. Another previous study generated detailed RNA-seq data from 63 samples for *C. elegans* across the life cycle, and identified large numbers of genes changing expression levels during early and late embryogenesis [5].

The time from hatching to first reproduction of many branchiopods is short [7]. Branchiopods exhibit an anamorphic mode of development [8], which involves multiple instars, gradually leading to the development of an adult form [9]. Planktonic water fleas of the genus *Daphnia* (Cladocera) are excellent model organisms for tracking transcriptomic patterns during development. *Daphnia* species are keystone components in freshwater ecosystems: they are principal grazers of phytoplankton and are themselves prey items for zooplanktivorous fish/invertebrate predators [10]. Water fleas are sensitive to a wide range of environmental stressors, such as pollution, pathogens, cyanobacterial toxins and predation [11]. In addition, *Daphnia* can be cultured in the laboratory as genetically uniform clonal lines through parthenogenesis [10], and thus provide ample material for analysis of transcriptomic changes in a single genotype throughout the course of development. When conditions are ideal (i.e., at 20 °C, under optimal food conditions), the parthenogenetic reproductive *D. magna* females deposit newly formed eggs into their brood pouch every 3–4 days [12]. The eggs go through 12 visually identifiable embryonic stages, and are then released from their mother to become free-swimming juveniles [13]. Juveniles, which are morphologically similar to adults, moult four (or more) times before closing the circle by releasing the first clutch of eggs into their brood pouch at around eight days of age [13]. The life cycle of *Daphnia* is phenotypically plastic. For example, individuals reach maturity at a smaller size when exposed to fish predation [14, 15]. Individuals can also start resting-stage production with a change in photoperiod [16, 17]. ﻿

Publicly available *Daphnia* genomes [18–20] offer new comparative tools with which to investigate gene regulation during developmental processes. However, transcriptomic studies of *Daphnia* species addressing changes across life-cycle stages are still few in number, with most attempts focusing on the response of genes to ecological challenges [21, 22]. Using a microarray platform, a previous study reported full-genome transcription profiling of *D. magna* life-cycle stages: many gene models showed differential transcription patterns across the developmental stages [23]. In particular, the embryonic stage of *D. magna* showed the highest number of unique transcribed genes, mainly related to DNA, RNA and ribosome biogenesis, which were related to cellular proliferation and morphogenesis [23]. Although microarray technology continues to advance, it can only detect known sequences. On the other hand, transcriptomics, which has progressed dramatically in the past few years, can yield detailed information on the entire transcriptome [24].

In this study, we used RNA-Seq to sample the *D. mitsukuri* transcriptome at three developmental stages (early embryo, juvenile and parthenogenetic adult). *Daphnia mitsukuri*, a sister species to *D. pulex*, belongs to subgenus *Daphnia* and often occurs in East Asia [25, 26]. We sampled embryos and then every two days during the initial eight days of development (after birth), which happen to span one embryonic, three juvenile and one adult timepoint. We expected to identify different sets of genes underpinning the development of each developmental stage. In particular, we expected to detect highly expressed genes associated with cell proliferation at the embryonic stage [23], and the high expression of genes related to stress response at the adult stage because *Daphnia* individuals are continuously subject to a wide range of environmental stressors [10]. Our work provides new insights into gene expression patterns during *Daphnia* development and highlights important genes/pathways underpinning development throughout life cycle.

## Results

### Gene expression signatures at different developmental stages

**Fig. 1:**
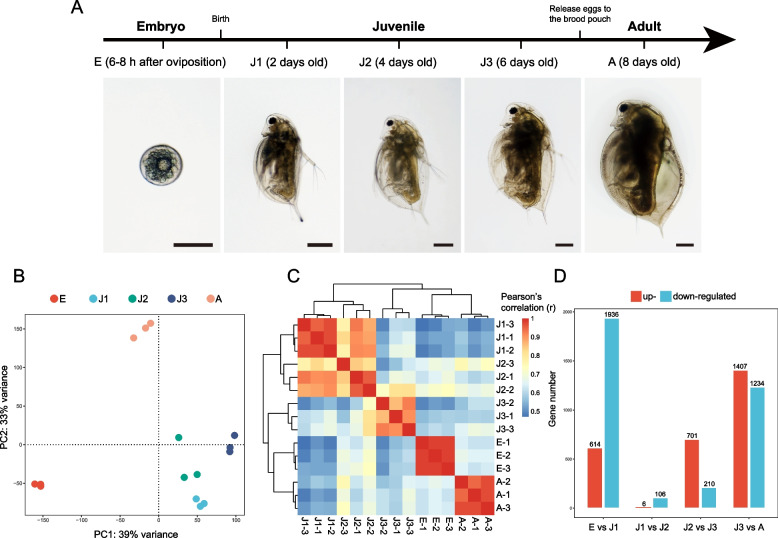
The gene expression signature of *Daphnia mitsukuri* during development. **A** Diagram representing the experimental design. Three developmental stages (three replicates of each time point) were selected: embryo (E: 6–8 h after oviposition); juvenile (J1: 2 days old after birth; J2: 4 days old; J3: 6 days old), and adult (A: 8 days old). Images of the studied developmental stages, scale bars in each image represent 200 μm. **B** Principal-component analysis of normalized gene expression counts for *D. mitsukuri* samples from three developmental stages. The plot of the first two principal components is shown. **C** Heat map of hierarchical clustering performed on Pearson correlations, calculated using normalized gene expression counts, between each pair of samples from three developmental stages (embryo, juvenile and adult). **D** Numbers of up- and down-regulated genes based on adjacent pairs of age groups. A gene with FDR-corrected *P*-value < 0.05 and FC ≥ 6 or was considered as up-regulated and a gene with FDR-corrected *P*-value < 0.05 and FC ≤ -6 was considered as down-regulated

We aimed to characterize the transcriptomic dynamics of parthenogenetic female *Daphnia mitsukuri* at three developmental stages (embryo, juvenile and adult: Fig. 1A). After trimming, an average of ~ 22 million reads per sample were obtained (Table S1), ~ 80% of which were mapped to only a single locus in the high-quality chromosome-level *D. mitsukuri* genome [20]. We detected the expression of 12,670 genes, out of 14,039 predicted genes in the *D. mitsukuri* genome.

In order to explore the temporal expression patterns of genes in our data sets, principal component analysis (PCA) was performed (Fig. 1B). The first two principal components (PC1 and PC2) explained 39% and 33% of the variation among samples, respectively. The gene expression profiles of the three biological replicates were close to each other in every case, and the samples collected at different developmental timepoints were separated by two principal components (Fig. 1B). Consistently, Pearson correlation analysis for all pairs of RNA-seq samples showed clear separations among the developmental timepoints (Fig. 1C). The clustering of gene expression showed close similarity among J1, J2 and J3, but a clear divergence between E (and A) and any other developmental stage. The enriched GO terms of top 500 genes that drive the separation in the PCA (based on all the gene in our data sets) see Supplementary Materials.

Differential gene expression between any two adjacent developmental stages was apparent. Substantial changes were detected from E to J1 (614 upregulated genes and 1,936 downregulated genes), and from J3 to A (1,407 upregulated genes and 1,234 downregulated genes; Fig. 1D). In contrast, there were few differences between J1 and J2 (6 upregulated genes and 106 downregulated genes; Fig. 1D). The GO terms enriched by up- and down-regulated genes based on adjacent pairs of age groups are listed in Table S2.

### Co-expression network

**Fig. 2:**
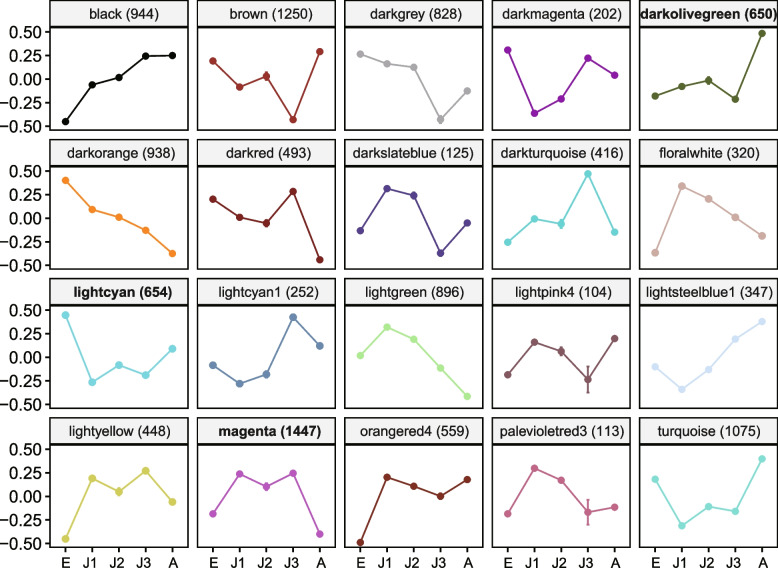
Module eigengene values of 20 distinct co-expression modules across all developmental stages. The vertical axes indicate module eigengenes. The horizontal axes indicate the stages. Error bars indicate SD of three biological replicates. The modules are named according to their assigned color, and the number of genes residing in each module is given in parentheses. Modules exhibiting the strongest positive correlations with particular developmental stages are labeled in bold

By applying weighted gene co-expression network analysis (WGCNA; based on expression data from all developmental stages), we identified a total of 20 modules that ranged in size from 104 genes (“lightpink4” module) to 1,447 genes (“magenta” module; Fig. 2 & Fig. S1). Each module from WGCNA represents a set of genes sharing a highly similar expression pattern during *D. mitsukuri* development (Fig. 2). We detected modules that exhibited a strong positive correlation with each developmental timepoint (Fig. S2): embryo (“lightcyan” module: *r* = 0.87, false discovery rate (FDR) corrected *P*-value = 5e-04; “darkorange” module: *r* = 0.78, FDR-corrected *P*-value = 0.01), J1 (no correlated modules), J2 (no correlated modules), J3 (“lightcyan1” module: *r* = 0.82, FDR-corrected *P*-value = 0.003; “darkturquoise” module: *r* = 0.91, FDR-corrected *P*-value = 4e-05), adult (“lightsteelblue1” module: *r* = 0.73, FDR-corrected *P*-value = 0.03; “darkolivegreen” module: *r* = 0.94, FDR-corrected *P*-value = 4e-06; “turquoise” module: *r* = 0.77, FDR-corrected *P*-value = 0.01).

### Specific highly expressed genes at the embryonic developmental stage

**Fig. 3:**
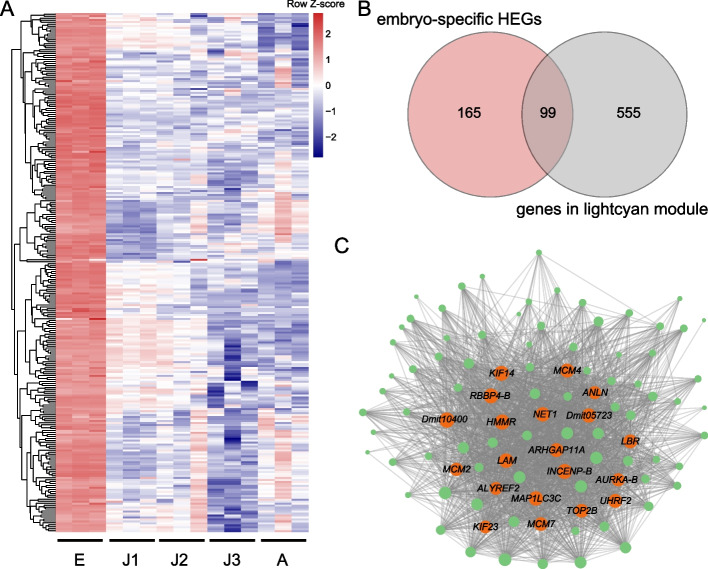
Expression pattern of genes related to embryonic development of *Daphnia mitsukuri*. **A** Expression heatmaps of embryo-specific highly expressed genes (HEGs) in *D. mitsukuri*. **B** Venn diagram showing the overlap between embryo-specific HEGs and genes in the “lightcyan” module. **C** Co-expression network of embryo-specific HEGs that were also found in the “lightcyan” module. Circle sizes represent the relative importance of each gene in the network. Orange dots represent the top 20 most-connected genes in this network, and other genes are represented by green dots

At the embryonic stage, we identified 264 highly expressed genes (HEGs; which have significantly higher expression at one timepoint than at others, with a fold change ≥ 6 and FDR-corrected *P*-value < 0.05) (Fig. 3A). GO enrichment analysis showed that these genes were involved in cell proliferation (e.g., GO terms “DNA helicase activity” and “DNA replication initiation”) and cell differentiation (e.g., GO terms “multicellular organism development”, “cell differentiation” and “neuron differentiation”; Fig. S3). Additionally, these HEGs were enriched in 12 KEGG pathways, five of which were associated with cell proliferation and cell differentiation (Fig. S4). Also, we identified the “lightcyan” module as having the strongest positive association with the embryonic stage (*r* = 0.87, FDR-corrected *P*-value < 0.001; Fig. S2). There was a significant overlap between embryo-specific HEGs and genes in the “lightcyan” module, with 99 overlapping genes (Fisher’s exact test; *P* < 2.2e-16; Table S3 & Fig. 3B). These genes were overrepresented in GO terms associated with cell proliferation (e.g., DNA replication initiation and replication fork; Fig. S5). An expression network of these overlapping genes identified the top 20 (hub genes) with the highest degree of connectivity (Fig. 3C). Interestingly, more than half of the hub genes were putatively involved in cell proliferation, including *KIF14*, *MCM7*, *MCM4* and *MCM2* (Fig. 3C & Table S4).

At the embryonic stage, we also looked at genes that belong to the “lightcyan” module but were not HEGs. These genes were involved in “cell division” (e.g. *CKS1B*, *CDC5*, *WLS*, *CDC6*, *ORC1*) and “DNA replication initiation” (e.g. *ORC5*, *ORC1*, *MCM10*, *MCM6*; Fig. S6). Among HEGs that did not belong to the “lightcyan” module were those involved in “Notch signaling pathway” (e.g. *NOTCH1*, *DLL1*, *JAG1*) and “multicellular organism development” (e.g. *SCR*, *UBX*, *OTX5*, *Hedgehog*, *DKK3*; Fig. S7).

### Specific highly expressed genes at the juvenile developmental stages

**Fig. 4:**
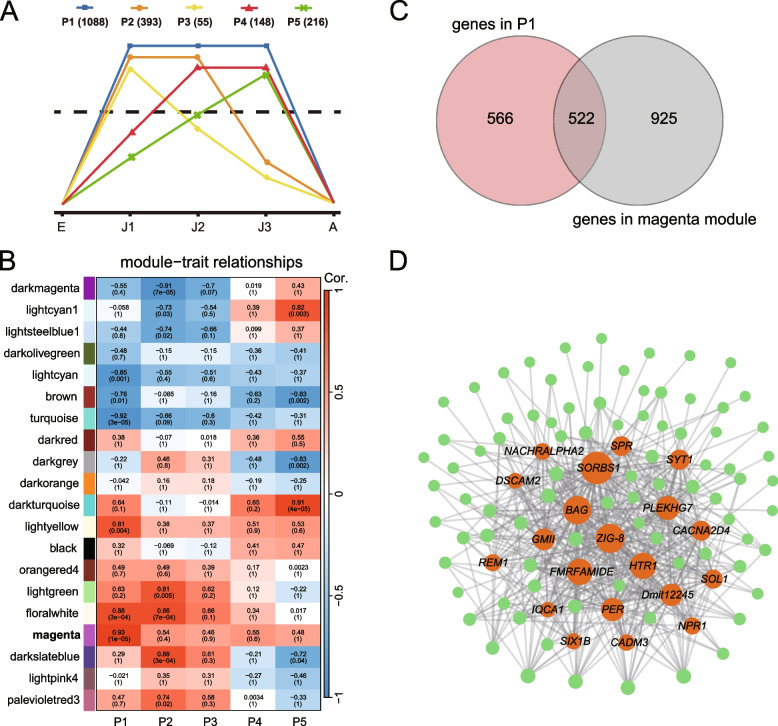
Gene expression patterns involved in the juvenile development of *Daphnia mitsukuri*. **A** Gene expression patterns of genes highly expressed at the juvenile stages in *D. mitsukuri*. The numbers in parentheses after each pattern indicates the number of genes that exhibit that pattern (P1-P5). **B** Correlations between module eigengenes and developmental stages in *D. mitsukuri* according to the WGCNA analysis. The numbers within the heat map represent correlations and FDR-corrected *P*-values (in parentheses; red, positively correlated, blue, negatively correlated) for the module-pattern associations. **C** Venn diagram showing the overlap between genes in P1 and genes in the “magenta” module. **D** Co-expression network of P1 genes that were found in “magenta” module. Edges with weight > 0.2 are plotted. Circle sizes represent the relative importance of each gene in the network. Orange dots represent the top 20 most-connected genes in this network and other genes are represented by green dots

A total of 1,900 stage-specific HEGs were identified at the juvenile developmental stages (J1–J3: Fig. 4A). These fell into five distinct patterns of expression (labeled P1–P5) from embryonic to adult development (Fig. 4A). Gene expression pattern P1 (high expression across J1, J2 and J3) was by far the most common pattern observed (1,088 out of 1,900 HEGs). GO enrichment analysis showed that genes in P1 were involved in visual perception (e.g., GO terms “phototransduction” and “detection of visible light”), chemosensory perception (e.g., GO terms “sensory perception of bitter taste” and “sweet taste receptor activity”) and neurotransmission (e.g., GO terms “neuropeptide signaling pathway” and “chemical synaptic transmission”; Fig. S8). The GO terms enriched by genes in P2-P5 are listed in Table S5. Additionally, genes in P1 were enriched in 39 KEGG pathways, including “phototransduction”, “taste transduction” and “neuroactive ligand-receptor interaction” (Fig. S9). Also, we found that the “magenta” module has the highest positive correlation with the gene expression pattern P1 (*r* = 0.87, FDR-corrected *P*-value < 0.001; Fig. 4B). There was a significant overlap between genes in P1 and the “magenta” module, resulting in 522 overlapping genes (Fisher’s exact test; *P* < 2.2e-16; Table S6 & Fig. 4C). These genes were enriched in GO terms related to chemosensory perception (e.g., “sensory perception of bitter taste”) and neurotransmission (e.g., “neuropeptide signaling pathway”; Fig. S10). An expression network of these overlapping genes identified the top 20 (hub genes) with the highest degree of connectivity (Fig. 4D). Seven of these were putatively involved in neurotransmission, including *ZIG-8*, *HTR1*, *SYT1*, *SOL1* (Fig. 4D & Table S7).

At the juvenile developmental stages, we looked at genes that belonged to the “magenta module” but not in P1. These genes were involved in “phospholipid catabolic process” and “intracellular signal transduction” (Fig. S11). We also examined genes in P1 but not in the “magenta” module. These genes were involved in “G protein-coupled receptor signaling pathway” (e.g. *TRHR*, *MAChR-A*, *SIFAR*, *CCHA1-R*, *HRH1*), “phototransduction” (e.g. *BCRH2*, *RHO*, *SCOP1*, *OP2*, *UVOP*; Fig. S12).

### Specific highly expressed genes at the adult developmental stage

**Fig. 5:**
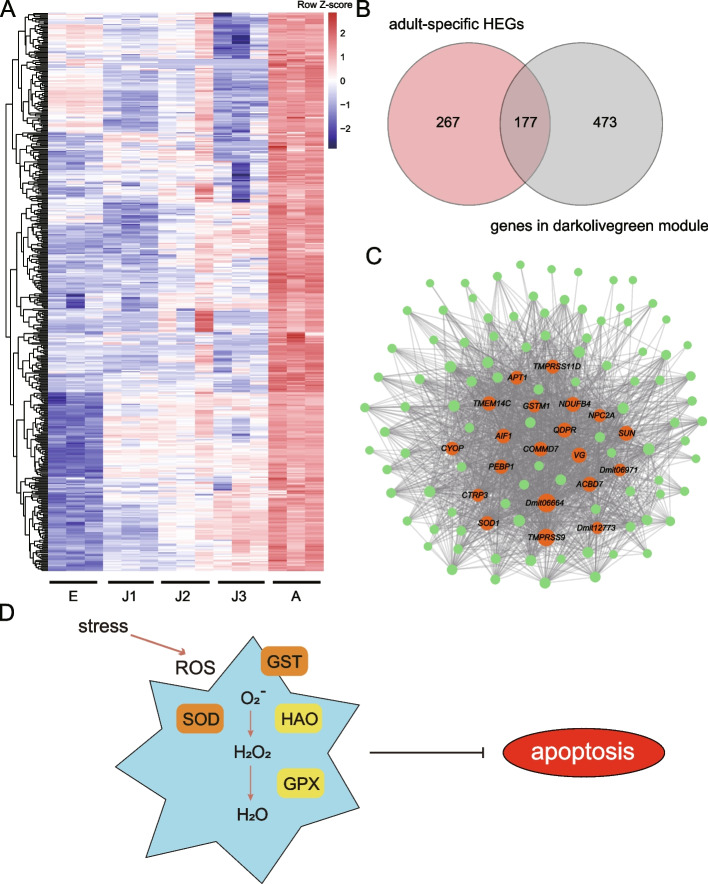
Expression pattern of genes associated with the development of adults of *Daphnia mitsukuri*. **A** Expression heatmaps of adult-specific highly expressed genes (HEGs) in *D. mitsukuri*. **B** Venn diagram showing the overlap between adult-specific HEGs and genes in the “darkolivegreen” module. **C** Co-expression network of adult-specific HEGs that are also found in the “darkolivegreen” module. Circle sizes represent the relative importance of each gene in the network. Orange dots represent the top 20 most-connected genes (hub genes) in this network and other genes are represented by green dots. **D** Candidate genes (hub genes, orange box; HEGs, yellow box) involved in antioxidative defensive system

At the adult developmental stage, we detected a total of 444 stage-specific HEGs (Fig. 5A). Notably, we observed high expression levels of *HAO* and *GPX*, encoding two important antioxidative enzymes (Fig. 5D). GO enrichment analysis showed that HEGs were associated with cuticle formation (e.g., GO terms “structural constituent of cuticle”, “chitin binding” and “chitin metabolic process”), lipid transport (e.g., GO terms “lipid transporter activity”, “lipid binding”) and stress response (e.g., GO terms “defense response to other organism” and “removal of superoxide radicals”; Fig. S13). KEGG analysis showed that these adult-specific HEGs were enriched in “biosynthesis of secondary metabolites”. We found that the “darkolivegreen” module had the highest positive association (r = 0.94, FDR-corrected *P*-value < 0.001) with the adult stage (Fig. S2). There was also a significant overlap between adult-specific HEGs and genes in the “darkolivegreen” module, resulting in 177 overlapping genes (Fisher’s exact test; *P* < 2.2e-16; Fig. 5B & Table S8). These genes were enriched in GO terms related to proteolysis, lipid transport, cuticle formation and stress response (Fig. S14). An expression network of these overlapping genes identified the top 20 (hub genes) with the highest degree of connectivity (Fig. 5C). These hub genes were involved in various activities including proteolysis, lipid transporter activity, and ATP synthase. Interestingly, we detected three hub genes (i.e., *SOD1*, *GSTM1*, and *QDPR*) associated with activities against oxidative stress (Fig. 5C & Table S9).

At the adult stage, we looked at genes that belonged to the “darkolivegreen” module but were not HEGs. These genes were associated with “mitochondrial electron transport”, “electron transport chain” and “mitochondrion” (Fig. S15). We also looked at HEGs that did not belong to the “darkolivegreen” module. These were involved in “defense response to other organism” and “structural constituent of cuticle” (Fig. S16). Additionally, the expression changes of genes related with key functions underpinning the development and moulting (i.e. DNA replication, differentiation, sensory perception, cuticle formation and stress responses) see Supplementary Materials.

### Highly expressed genes and expanded or specific gene families of the genus Daphnia

For each developmental stage, we looked at the HEGs that also occurred in expanded gene families identified in *Daphnia* genomes [20]. There was a significant overlap (*P* = 0.026) between expanded gene families of *Daphnia* species and HEGs (P1 genes) at the juvenile stages, but this was not the case at the embryonic stage (*P* = 0.99) nor at the adult developmental stage (*P* = 0.33; Table S20). Specifically, at the juvenile stages, we detected 54 P1 genes that also belong to the expanded gene families of *Daphnia* species (Table S11). GO enrichment analysis showed that these genes were involved in “G protein-coupled receptor signaling pathway”, “phototransduction”, “visual perception” and “postsynapse” (Fig. S17). Notably, nine out of these 54 HEGs encoded rhodopsin which functions as the primary photoreceptor molecule of vision, and two of them encoded ionotropic glutamate receptor which mediates fast synaptic transmission in the central nervous system (Table S11).

We also looked at expression of *Daphnia*-specific gene families at each developmental stage. There is a significant overlap (*P* = 0.00045) between *Daphnia*-specific gene families [20] and HEGs at the adult stage, but this was not the case at the embryonic stage (*P* = 0.21) nor at the juvenile stages (*P* = 0.94; Table S12). At the adult stage, we detected 50 HEGs belonging to *Daphnia*-specific gene families (Table S13). These genes were enriched in three GO terms “oxygen carrier activity”, “oxygen binding” and “oxygen transport”.

## Discussion

Transitions between developmental stages are always accompanied by substantial alterations in gene expression [2]. We investigated gene expression patterns of *D. mitsukuri* across different developmental stages, including embryo, juvenile (three timepoints), and adult. Our findings revealed clear separations among these developmental stages in *D. mitsukuri*, consistent with a previous study for *D. magna* using microarray technology [23]. We identified sets of highly expressed genes (HEGs) underpinning each of the three developmental stages of *D. mitsukuri*. Overall, our result has revealed gene -expression patterns during *Daphnia* development and identified important candidate genes/pathways underpinning development.

Our investigation revealed that a considerable number of HEGs at the embryonic stage were related to cell proliferation, cell differentiation and morphogenesis. Regulation of cell proliferation is mainly controlled by two waves of transcription that occur at the onset of DNA replication at the interphase S-phase and during mitosis at the M-phase [27]. We found that several hub genes (e.g. *MCM2*, *MCM4 and MCM7*) at the embryonic stage were closely associated with DNA replication. MCM2-7 proteins are evolutionarily conserved in all eukaryotes, being key components of the pre-replication complex that forms at the origin of DNA replication [28]. Upregulation of MCM genes is observed in proliferating cells [29]. For example, maternally supplied MCM proteins are abundant in the early embryos of *Drosophila* [30], and mutations of the MCM genes inhibited proliferation of cells and further caused an apparent prolongation of S phase in the embryos of *Drosophila* [31]. Also, we detected several hub genes (e.g. *KIF14*; with the highest degree of connectivity in the network) at the embryonic stage that are closely associated with mitosis. The expression of *KIF14* plays an essential role in cytokinesis/mitosis: depletion of this gene results in incomplete cytokinesis/mitosis and multi-nucleation [32, 33]. For example, downregulation of *KIF14* suppresses cell proliferation, and subsequently induces apoptosis in various human tissues [34, 35]. Loss of gene *KIF14* leads to cytokinesis and developmental defects in *Drosophila* and to microcephaly and growth retardation in mice [36]. Overall, our findings strongly suggest that cell-proliferation processes are predominant during the early embryonic stage of *D. mitsukuri*, as observed during embryogenesis in other species, for example *Drosophila melanogaster* [37], *Caenorhabditis elegans* [5] and *D. magna* [23]. 

Previous studies found that many differentially transcribed genes at the embryonic stage of *D. magna* were involved in signaling pathway (including Wnt and Notch signaling pathways) [23]. In line with these previous findings, some HEGs at the embryonic stage of *D. mitsukuri* play crucial roles in evolutionarily conserved signaling pathways, including Wnt-1 and Wnt-2b in the Wnt signaling pathway, *NOTCH1*, *DLL1* and *JAG1* in the Notch signaling pathway, and *Hedgehog* in the Hedgehog signaling pathway. In *Drosophila*, the expression of *Wnt* and *Hedgehog* genes are initiated shortly after egg fertilization, with reciprocal regulation stabilizing their expression [38]. These findings suggest an essential role of the signaling pathways in the embryonic development of arthropod species. Here, another set of HEGs at the embryonic stage, also highly expressed in the embryonic stage of *D. magna* [23], was associated with structural morphogenesis. Some orthologs of these HEGs play an important role in morphogenesis for *Drosophila*; for example, mutation of *SLP* (expressed in the embryonic head) led to strong segmentation defects and a failure of head involution [39], and the mutation of *INX2* (expressed during the early embryonic stage) resulted in a feeding defect and a failure of proventriculus development [40]. It should be noted that our investigation only focused on gene expression at the early embryonic stage in *D. mitsukuri*. To gain a more comprehensive understanding of embryonic development of *Daphnia*, future studies should include more samples from the 12 visually identifiable embryonic stages [13].

*Daphnia* individuals are continuously subjected to a wide range of environmental stressors [10, 41]. Among these is the presence of predators, be they zooplanktivorous fishes or invertebrates [10]. Such predators release info-chemicals (kairomones) into the surrounding water that can be detected by *Daphnia* [42, 43] A most recent study has shown that Daphnia can accurately assess predation risks as a result of expansion of multiple gene families associated with chemoreception and vision [20]. Our results showed that HEGs at juvenile stages (i.e., J1, J2 and J3) were enriched in GO and KEGG terms related to chemosensory perception and visual perception. Interestingly, we found a significant overlap (54 genes) between expanded gene families of *Daphnia* species and HEGs at the juvenile stages. Of note are opsin genes, which were significantly expanded in the *Daphnia* genomes [18, 20]. Nine of these 54 HEGs belong to the *RHO* gene family that encode rhodopsin which is the primary photoreceptor in the visual signaling cascade [44]. A previous study showed that rhodopsin played an essential structural role in *Drosophila* photoreceptor development, and its mutation resulted in developmental defects in the photosensitive membranes [45]. Upregulation of this gene could enable *Daphnia* to enhance visual detection of predation risk [20]. Detection of potential environmental stressors via visual perception at the juvenile stages is likely critical to survival and fitness of *D. mitsukuri*.

Another set of enriched GO terms at the juvenile stages was related to neurotransmission. This GO term was also enriched by differentially transcribed genes at the juvenile stage in *D. magna* [23]. Notably, we found that the gene *ZIG-8* related to neurotransmission and showed a high degree of connectivity in the co-expression network. This gene plays a key role in the establishment of neuronal connectivity across bilaterians [46], such as *C. elegans* [47] and *D. melanogaster* [46]*.* Therefore, we assumed that the enhanced expression of genes associated with neurotransmission promote the neurodevelopment of juvenile *Daphnia*. This would help *Daphnia* to detect/respond to environmental stress.

Environmental stress triggers the increased production of reactive oxygen species (ROS), and subsequently results in an imbalance that can lead to cell and tissue damage [48]. Activation of antioxidative defensive systems is thus needed to protect cells from ROS-induced damage [49]. Here, we detected high expression of genes at the adult stage that were enriched in GO terms “glutathione metabolic process” and “removal of superoxide radicals”. We also identified three hub genes, *SOD1*, *GSTM1* and *QDPR*, which are associated with activities against oxidative stress. *GSTM1* [50] and *SOD1* [51] are critical antioxidant enzymes that can suppress apoptosis triggered by cellular stressors. For example, upregulation of *GST* and *SOD* help *D. magna* to cope with ultraviolet radiation-induced oxidative stress [52, 53]. Indeed, *SOD1* is expressed throughout the adult *Drosophila* lifespan [54], and the mutation of *SOD1* in *D. melanogaster* increases their sensitivity to hydrogen peroxide [55] and thus shortens their lifespan [56]. We also detected high expression levels of two genes at the adult stage, *HAO* and *GPX*, which encode antioxidant enzymes that protect cells from oxidative damage [57, 58]. This agrees with a previous study which found that differentially transcribed genes at the adult stage in *D. magna* [23] were enriched in GO terms related to stress response, including “response to stress” and “regulation of mRNA stability involved in response to stress”. High expression levels of genes associated with stress responses (regardless of the actual presence or absence of stress) in adults might activate the antioxidative defensive system, helping *Daphnia* to cope with both abiotic and biotic stimuli. Another set of HEGs at the adult stage in both *D. magna* [23] and *D. mitsukuri* (this study) were associated with structural constituents of cuticle, perhaps in line with the increased quantity of cuticle required at the adult stage in *Daphnia* [22]. The genome of *Daphnia* contains numbers of specific gene families [18], and we found a significant overlap between HEGs at the adult stage and *Daphnia*-specific gene families. These specific gene families might exhibit a high expression at the adult stage and are critical in responsive to ecological challenges.

Our data, covering three developmental stages of *Daphnia mitsukuri* from early embryo to parthenogenetic adult, provide new insights into the transcripts present in the whole animal at each stage. At the early embryonic stage, cell proliferation is the dominant activity, ensuring the necessary foundation for subsequent development. After emergence from the brood pouch, the high expression of genes at the juvenile stages associated with chemoreception and vision allows *Daphnia* to enhance detection of potential environmental risks, and high expression of genes in the adult that are associated with antioxidative defensive systems allows *Daphnia* to mount an efficient response to perceived environmental risks. While these data help to delineate gene expression dynamics of *Daphnia*, a larger sample size and additional timepoints are necessary to strengthen these findings. Future studies should also confirm our observations on gene expression at the experimental level.

## Materials and methods

### Daphnia developmental samples

We investigated the transcriptomic dynamics of *Daphnia* at three developmental stages: embryo (6–8 h after oviposition), juvenile (including three time points: J1 (2 days old after birth, second instar), J2 (4 days old, third instar) and J3 (6 days old, fourth instar) and adult (8 days old, fifth instar; Fig. 1A). Thus, we sampled embryos and every two days after birth, spanning one embryonic (E), three juvenile (J1-3) and one adult (A) timepoints. *Daphnia mitsukuri*, a taxonomically valid species with a wide distribution in East Asia, was used in this study [25, 26]. A single *D. mitsukuri* clone was collected from “Suzhou Pond” (DP; 31°23′ N, 121°41′ E) and maintained in the laboratory at moderate densities (~ 30 adults/jar) in 500 mL glass jars (with 450 mL COMBO medium [59]), at 20 °C under a 16:8 h light:dark cycle and fed three times per week with unicellular algae *Ankistrodesmus falcatus*. Four hundred adult females of *D. mitsukuri* were transferred from stock cultures into 40 jars (10 individuals per jar, 150 ml COMBO medium) to be the parents of the eggs/neonates used in this study. Embryos (~ 1,000) were collected 6–8 h after oviposition and randomly distributed among three Eppendorf tubes. We collected the embryos according to the procedure described in Mittmann et al. [13]. Briefly, each female (210 in total) was transferred to a petri dish in a small drop of medium. While fixing them by pinning the carapace facing the petri dish down with a blunt needle, the eggs were gently removed from underneath the carapace with a second blunt needle. For the later developmental stages, 900 neonates, which were emerged from the brood pouch within the previous 24 h from the remaining adult mothers, were randomly placed into 90 experimental jars (10 neonates per jar, 200 ml medium). Then, 36 jars at J1, 24 jars at J2, 18 jars at J3 and 12 jars at adult stage were randomly selected and divided into three Eppendorf tubes separately, representing three biological replicates per timepoint. As expected, we did not observe any male offspring during the experiment. The contents of each Eppendorf tube were homogenized and flash-frozen in liquid nitrogen.

### RNA isolation, library preparation and sequencing

Total RNA of each snap-frozen sample was extracted using the RNeasy Mini Kit (Qiagen, Valencia, CA) following the manufacturer’s instructions. RNA purification, including on-column DNA digestion, was performed using the RNeasy Kit (Qiagen, Hilden, Germany), according to the manufacturer’s protocol. The quality and quantity of the purified RNA were determined using a NanoDrop 2000c Spectrophotometer (Thermo, USA) and an RNA Nano Chip assay on an Agilent Bioanalyzer (RIN > 7.5 for all samples). Then, 10 µg of total RNA from each sample was used for RNA-seq library construction with a TruSeq RNA Sample Preparation Kit (Illumina), resulting in a total of 15 libraries (5_time points_ × 3_replicates_). The libraries were sequenced in a single lane on an Illumina Novaseq 6000 Platform with paired-end sequencing of 150 bp read length (Novogene, Tianjin, China).

### Quality control, read mapping and transcriptome analyses

Low-quality RNA-seq reads with a PHRED score < 25 were discarded and adapters were trimmed using TRIMGALORE (https://github.com/FelixKrueger/TrimGalore; parameters: -q 25 –phred33 –stringency 3 –fastqc). The STAR aligner [60] was used to map the clean reads to the chromosome-level *D. mitsukuri* genome [20] with default parameters (1-pass mapping mode). The assembly was 145 Mb in 173 scaffolds (across 12 chromosomes) with 55.3 Mb of repeated sequences and 14,039 predicted genes. Gene ontology (GO) annotations of *D. mitsukuri* genes were created by combining NR and InterPro annotations using BLAST2GO CLI [61], and pathway assignments of *D. mitsukuri* genes were performed based on the Kyoto Encyclopedia of Genes and Genomes (KEGG) database via online KEGG Automatic Annotation Server (KAAS) [62] (Please see Zhang et al. [20] for details). More than 93% of protein-coding genes in the *D. mitsukuri* genome have homologs in one or more public databases (NCBI NR, UniProt, KOG, KEGG, Pfam and Gene ontology; Table S14). Gene counts were created using HTseq-count v 0.12.4 (parameters: -f bam -r pos -s no -i gene_id) [63]. We subsequently performed differential expression analysis based on the negative binomial distribution in the R package DEseq2 [64], with raw counts as input. The transformed counts following variance-stabilizing transformation in DESeq2 were used to perform PCA with all expressed genes (ntop = 12,670). Additionally, the Pearson correlations between biological replicates were calculated using the R function “cor”, based on the normalized count data of all expressed genes (*n* = 12,670). A heat map of hierarchical clustering was constructed on Euclidean distance in the R package “pheatmap”, calculated using normalized gene expression counts.

### Analysis of development-dependent gene expression

Genes that were differentially expressed between adjacent pairs of developmental timepoints were identified in DESeq2. Comparisons were made using the older developmental timepoint as denominator, that is, embryo versus J1, J1 versus J2, J2 versus J3, and J3 versus adult. A gene with fold change ≥ 6 and false discovery rate (FDR) corrected *P*-value < 0.05 was considered as up-regulated during that developmental timepoint bracket. A gene with FC ≤ -6 and FDR-corrected *P*-value < 0.05 was considered as down-regulated.

### Identification of stage-specific highly expressed genes (HEGs)

To identify stage-specific HEGs, comparisons were made between any given developmental timepoint (treated as a group) and all other timepoints combined (treated as another group). A gene with FC ≥ 6 and FDR-corrected *P*-value < 0.05 was considered to be highly expressed during that developmental timepoint. We defined five patterns of gene expression at the juvenile developmental timepoints. They are labeled as patterns P1–P5, as follows: P1, genes highly expressed at J1, J2, and J3 compared with E, A; P2, genes highly expressed at J1, J2 compared with E, J3 and A; P3, genes highly expressed at J1, compared with E, J2, J3 and A; P4, genes highly expressed at J2 and J3, compared with E, J1 and A; P5, genes highly expressed at J3 compared with E, J1, J2 and A. Gene ontology (GO) and KEGG enrichment analyses were performed for HEGs at different developmental stages separately, using the ClusterProfiler package [65] with FDR correction in R. Significantly enriched GO and KEGG terms were identified with an FDR-corrected *P*-value of ≤ 0.05. Please note, in most non-model organisms, such as *D. mitsukuri*, gene-set enrichment analysis might have limited power, largely because functional annotations are only available for a fraction of the genes analyzed.

### Co-expression analysis

Only genes with the sum of DESeq2-normalized read counts of at least 10 were selected for the unsupervised weighted gene co-expression network analysis (WGCNA). A total of 12,061 genes passed this filtering criterion. Then, log2-transformed DESeq2-normalized counts served as input for the WGCNA [66] in R, using the function blockwiseModules to create a signed network of a Pearson correlated matrix. Here, a soft power threshold of 9 was chosen since this was the lowest power needed to reach scale-free topology (R^2^ = 0.92). Module detection was performed with default parameters, and the minimal module size was set to 30 genes. Subsequently, highly correlated modules were merged by using a cut height of 0.2, reducing the number of modules from 56 to 20. For each module, we calculated the expression profile of the module eigengene which is defined as the first principal component of the module’s expression data. We then applied module eigengene values to test for associations between module expression and developmental stages for each module (Pearson correlation; *cor* function in WGCNA). *P*-values for the correlation were computed using a Student’s asymptotic test (*corPvalueStudent* function in WGCNA), and subsequently corrected using the FDR method. For each developmental stage, we looked at the HEGs also found in a WGCNA module that exhibited the highest positive correlation with that specific stage. Venn diagrams were constructed to depict the number of HEGs also found in WGCNA modules. Fisher’s exact test was applied to determine whether these HEGs significantly overlapped with a WGCNA module (12,061 genes mentioned above were included in this analysis). Similar GO enrichment analysis for the overlapping genes was performed with FDR-corrected *P*-value of ≤ 0.05. The topological overlap matrix of these genes was generated using exportNetworkToCytoscape function in WGCNA with an adjacency cutoff of > 0.2, and then was exported to Cytoscape v3.3 [67] to visualize weighted coexpression networks. The degree of connectivity of each gene, determined as the sum of the edge attributes of genes connected to it, reflects the node size. The higher the connectivity is, the stronger the biological function of the gene. The top 20 genes with the highest degree of connectivity were considered as hub genes.

### Comparison of HEGs and expanded or specific gene families of Daphnia

For each developmental stage, we looked at the shared genes of HEGs and expanded (or specific) gene families in *Daphnia* species. These expanded (or orphan) *Daphnia* gene families were retrieved from our previous study [20]. Fisher’s exact test was applied to determine whether the HEGs significantly overlapped with the expanded (or specific) gene families in *Daphnia* (a set of 12,670 genes with datable expression during the development of *D. mitsukuri* were included in this analysis). Similar GO enrichment analysis for the overlapping genes was performed with FDR-corrected *P*-value of ≤ 0.05.

### Supplementary Information

**Supplementary Material 1. **

## Data Availability

The RNA-seq data are available in the NCBI database under BioProject accession number PRJNA1002854.
