# Supplementary material for: RNA-seq analysis reveals changes in mRNA expression during development in Daphnia mitsukuri
Source: BMC Genomics. 2024 Mar 21;25:302. doi: 10.1186/s12864-024-10210-8 (PMC10958850; doi:10.1186/s12864-024-10210-8)

**RNA-seq analysis reveals changes in mRNA expression during development in *Daphnia mitsukuri***

Xiuping Zhang^1,#^, Wenwu Yang^1,#^, David Blair^2^, Wei Hu^1^ and Mingbo Yin^1*^

^1^MOE Key Laboratory for Biodiversity Science and Ecological Engineering, School of Life Science, Fudan University, Songhu Road 2005, Shanghai, China

^2^College of Marine and Environmental Sciences, James Cook University, Townsville Qld 4811, Australia

^#^Contributed equally.

^*^Corresponding author: Mingbo Yin; [yinm@fudan.edu.cn](mailto:yinm@fudan.edu.cn)

Keywords: development; temporal; transcriptomics; *Daphnia*; embryo; genes

**Results**

*Genes driving the separation in PCA*

We identified the top 500 genes that drive the separation in the PCA. GO enrichment analysis showed that these genes were associated with transition (e.g. GO terms “translation”, “cytoplasmic translation”, “mitochondrial translation”), ribosome (e.g. GO terms “cytosolic large ribosomal subunit”, “structural constituent of ribosome”; Fig. S18).

*Expression changes of genes underpinning fundamental functions in development of* Daphnia

Fifty GO terms, selected from enriched GO terms of HEGs or genes in WGCNA modules, were chosen to represent five key functions (i.e. DNA replication, differentiation, sensory perception, cuticle formation and stress responses) underpinning the development and moulting of *D. mitsukuri* (Fig. S19). We found that genes involved in DNA replication (including GO “DNA replication initiation”, “replication fork” and “DNA helicase activity”) showed a decreased level of expression from E to J1; genes involved in differentiation (including GO “multicellular organism development”, “cell differentiation” and “neuron differentiation”) decreased as development progressed (i.e. from E to A); genes related to sensory perception (including GO “visual perception”, “phototransduction” and “chemosensory behavior”) showed an elevated level of expression from E to J1; genes associated with cuticle formation (including GO “structural constituent of cuticle”, “chitin metabolic process” and “chitin binding”) showed an elevated level of expression from E to J1 and retained a high level of expression from J1 to A; genes related to response to environmental stress (including GO “defense response to other organism”, “superoxide dismutase activity” and “removal of superoxide radicals”) showed high expression levels at the adult stage.

Table S1. Sequencing and alignment information for all RNA-seq samples from three developmental stages: embryo (E: 6-8 h after oviposition); juvenile (J1: 2 days old after birth; J2: 4 days old; J3: 6 days old), and adult (A: 8 days old).

| Development stage | Sample | Raw | Filtered | Filtered/Raw | Unique mapped | Unique/Filtered |
| --- | --- | --- | --- | --- | --- | --- |
| E | mit-E-1 | 24,631,208 | 24,563,100 | 99.7% | 21,467,775 | 87.4% |
|  | mit-E-2 | 22,437,469 | 22,362,985 | 99.7% | 19,581,699 | 87.6% |
|  | mit-E-3 | 26,754,165 | 26,670,670 | 99.7% | 23,393,776 | 87.7% |
| J1 | mit-J1-1 | 21,403,765 | 21,302,911 | 99.5% | 18,803,399 | 88.3% |
|  | mit-J1-2 | 22,260,551 | 22,142,879 | 99.5% | 19,308,336 | 87.2% |
|  | mit-J1-3 | 20,351,979 | 20,245,580 | 99.5% | 17,503,059 | 86.5% |
| J2 | mit-J2-1 | 21,185,432 | 21,089,433 | 99.5% | 18,529,817 | 87.9% |
|  | mit-J2-2 | 22,021,646 | 21,875,999 | 99.3% | 18,740,019 | 85.7% |
|  | mit-J2-3 | 23,711,909 | 23,614,983 | 99.6% | 19,655,432 | 83.2% |
| J3 | mit-J3-1 | 21,567,944 | 21,490,337 | 99.6% | 15,187,796 | 70.7% |
|  | mit-J3-2 | 21,672,593 | 21,580,959 | 99.6% | 15,216,390 | 70.5% |
|  | mit-J3-3 | 22,124,986 | 22,027,617 | 99.6% | 15,868,293 | 72.0% |
| A | mit-A-1 | 23,565,249 | 23,474,315 | 99.6% | 19,358,495 | 82.5% |
|  | mit-A-2 | 21,225,141 | 21,140,485 | 99.6% | 17,301,633 | 81.8% |
|  | mit-A-3 | 21,687,965 | 21,603,041 | 99.6% | 17,627,589 | 81.6% |

Table S2. GO enrichment terms of up- and down-regulated genes based on adjacent pairs of age groups (i.e. E vs. J1, J1 vs. J2, J2 vs. J3, J3 vs. A) in the development of *Daphnia mitsukuri*.

| Gene set | GO id | GO terms | FDR-corrected *P*-value | Number of genes |
| --- | --- | --- | --- | --- |
| Up-regulated in E vs. J1 | GO:0004298 | threonine-type endopeptidase activity | 1.44496E-10 | 11 |
|  | GO:0000398 | mRNA splicing, via spliceosome | 1.68056E-05 | 14 |
|  | GO:0006270 | DNA replication initiation | 1.83314E-05 | 9 |
|  | GO:0071011 | precatalytic spliceosome | 8.46489E-05 | 8 |
|  | GO:0071013 | catalytic step 2 spliceosome | 0.000151101 | 10 |
|  | GO:0051301 | cell division | 0.000225399 | 8 |
|  | GO:0006334 | nucleosome assembly | 0.000727339 | 7 |
|  | GO:0003677 | DNA binding | 0.002158683 | 35 |
|  | GO:0042575 | DNA polymerase complex | 0.002158683 | 6 |
|  | GO:0005689 | U12-type spliceosomal complex | 0.002852724 | 5 |
|  | GO:0003678 | DNA helicase activity | 0.003844163 | 6 |
|  | GO:0046983 | protein dimerization activity | 0.014173285 | 13 |
|  | GO:0005657 | replication fork | 0.014173285 | 9 |
|  | GO:0006511 | ubiquitin-dependent protein catabolic process | 0.014173285 | 11 |
|  | GO:0019013 | viral nucleocapsid | 0.029858167 | 7 |
|  | GO:0003723 | RNA binding | 0.03624043 | 27 |
| Down-regulated in E vs. J1 | GO:0007187 | G protein-coupled receptor signaling pathway, coupled to cyclic nucleotide second messenger | 5.46466E-22 | 55 |
|  | GO:0004930 | G protein-coupled receptor activity | 2.42343E-17 | 51 |
|  | GO:0007218 | neuropeptide signaling pathway | 4.33866E-16 | 30 |
|  | GO:0007186 | G protein-coupled receptor signaling pathway | 1.97529E-13 | 46 |
|  | GO:0005887 | integral component of plasma membrane | 1.63899E-11 | 61 |
|  | GO:0034220 | ion transmembrane transport | 1.62418E-10 | 26 |
|  | GO:0005576 | extracellular region | 1.15922E-08 | 56 |
|  | GO:0007602 | phototransduction | 1.15922E-08 | 12 |
|  | GO:0008061 | chitin binding | 3.84526E-08 | 39 |
|  | GO:0005886 | plasma membrane | 5.26591E-08 | 57 |
|  | GO:0005230 | extracellular ligand-gated ion channel activity | 6.97534E-08 | 14 |
|  | GO:0042302 | structural constituent of cuticle | 6.97534E-08 | 30 |
|  | GO:0004888 | transmembrane signaling receptor activity | 2.36749E-07 | 17 |
|  | GO:0008020 | G protein-coupled photoreceptor activity | 3.51824E-07 | 13 |
|  | GO:0009584 | detection of visible light | 1.12934E-06 | 11 |
|  | GO:0005615 | extracellular space | 1.22906E-06 | 37 |
|  | GO:0007601 | visual perception | 1.6812E-06 | 12 |
|  | GO:0004983 | neuropeptide Y receptor activity | 3.15384E-06 | 11 |
|  | GO:0007268 | chemical synaptic transmission | 7.49803E-06 | 29 |
|  | GO:0006030 | chitin metabolic process | 1.11936E-05 | 28 |
|  | GO:0020037 | heme binding | 1.59659E-05 | 35 |
|  | GO:0005506 | iron ion binding | 2.54231E-05 | 29 |
|  | GO:0004252 | serine-type endopeptidase activity | 3.30957E-05 | 36 |
|  | GO:0004197 | cysteine-type endopeptidase activity | 0.000112667 | 12 |
|  | GO:0010469 | regulation of signaling receptor activity | 0.000112667 | 15 |
|  | GO:0005249 | voltage-gated potassium channel activity | 0.000162147 | 10 |
|  | GO:0005764 | lysosome | 0.000224987 | 11 |
|  | GO:0008076 | voltage-gated potassium channel complex | 0.000224987 | 11 |
|  | GO:0060079 | excitatory postsynaptic potential | 0.000224987 | 16 |
|  | GO:0006508 | proteolysis | 0.000224987 | 71 |
|  | GO:0016020 | membrane | 0.000309049 | 82 |
|  | GO:0004672 | protein kinase activity | 0.000500962 | 28 |
|  | GO:0030054 | cell junction | 0.000862743 | 10 |
|  | GO:0035725 | sodium ion transmembrane transport | 0.001022509 | 13 |
|  | GO:0005516 | calmodulin binding | 0.001092897 | 9 |
|  | GO:0055085 | transmembrane transport | 0.001127689 | 42 |
|  | GO:0007166 | cell surface receptor signaling pathway | 0.001127689 | 17 |
|  | GO:0005234 | extracellularly glutamate-gated ion channel activity | 0.00208633 | 12 |
|  | GO:0051603 | proteolysis involved in protein catabolic process | 0.002957958 | 9 |
|  | GO:0043005 | neuron projection | 0.003993195 | 8 |
|  | GO:0034765 | regulation of ion transmembrane transport | 0.003993195 | 8 |
|  | GO:0016998 | cell wall macromolecule catabolic process | 0.004286268 | 11 |
|  | GO:0035235 | ionotropic glutamate receptor signaling pathway | 0.004481605 | 20 |
|  | GO:0043190 | ATP-binding cassette (ABC) transporter complex | 0.005037633 | 7 |
|  | GO:0098793 | presynapse | 0.006145067 | 8 |
|  | GO:0071805 | potassium ion transmembrane transport | 0.007649498 | 13 |
|  | GO:0005272 | sodium channel activity | 0.008669488 | 10 |
|  | GO:0045202 | synapse | 0.009076593 | 8 |
|  | GO:0016042 | lipid catabolic process | 0.009076593 | 8 |
|  | GO:0005184 | neuropeptide hormone activity | 0.01078211 | 6 |
|  | GO:0007165 | signal transduction | 0.01101382 | 39 |
|  | GO:0005975 | carbohydrate metabolic process | 0.012023756 | 25 |
|  | GO:0045211 | postsynaptic membrane | 0.01294448 | 8 |
|  | GO:0051260 | protein homooligomerization | 0.017897055 | 6 |
|  | GO:0004970 | ionotropic glutamate receptor activity | 0.018114405 | 20 |
|  | GO:0005544 | calcium-dependent phospholipid binding | 0.018896506 | 7 |
|  | GO:0016705 | oxidoreductase activity, acting on paired donors, with incorporation or reduction of molecular oxygen | 0.021039632 | 14 |
|  | GO:0004568 | chitinase activity | 0.021039632 | 9 |
|  | GO:0098794 | postsynapse | 0.021039632 | 9 |
|  | GO:0006032 | chitin catabolic process | 0.021039632 | 9 |
|  | GO:0080019 | fatty-acyl-CoA reductase (alcohol-forming) activity | 0.025247558 | 6 |
|  | GO:0010025 | wax biosynthetic process | 0.025247558 | 6 |
|  | GO:0035336 | long-chain fatty-acyl-CoA metabolic process | 0.025247558 | 6 |
|  | GO:0043025 | neuronal cell body | 0.026910621 | 9 |
|  | GO:0005509 | calcium ion binding | 0.027871012 | 39 |
|  | GO:0004016 | adenylate cyclase activity | 0.033898386 | 6 |
|  | GO:0004383 | guanylate cyclase activity | 0.033898386 | 6 |
|  | GO:0030198 | extracellular matrix organization | 0.033898386 | 7 |
|  | GO:0050804 | modulation of chemical synaptic transmission | 0.033898386 | 5 |
|  | GO:0006171 | cAMP biosynthetic process | 0.033898386 | 6 |
|  | GO:0006182 | cGMP biosynthetic process | 0.033898386 | 6 |
|  | GO:0046039 | GTP metabolic process | 0.033898386 | 6 |
|  | GO:0009986 | cell surface | 0.037297805 | 8 |
|  | GO:0004497 | monooxygenase activity | 0.039948511 | 9 |
|  | GO:0052689 | carboxylic ester hydrolase activity | 0.039959306 | 10 |
|  | GO:0016788 | hydrolase activity, acting on ester bonds | 0.047135592 | 6 |
|  | GO:0004114 | 3',5'-cyclic-nucleotide phosphodiesterase activity | 0.047135592 | 5 |
|  | GO:0022848 | acetylcholine-gated cation-selective channel activity | 0.047135592 | 5 |
|  | GO:0048488 | synaptic vesicle endocytosis | 0.047135592 | 5 |
| Up-regulated in J1 vs. J2 | GO:0005576 | extracellular region | 0.00043443 | 3 |
|  | GO:0008061 | chitin binding | 0.005930813 | 2 |
|  | GO:0015035 | protein-disulfide reductase activity | 0.025245609 | 1 |
|  | GO:0004568 | chitinase activity | 0.025245609 | 1 |
|  | GO:0006032 | chitin catabolic process | 0.025245609 | 1 |
|  | GO:0016998 | cell wall macromolecule catabolic process | 0.025245609 | 1 |
|  | GO:0006457 | protein folding | 0.036967928 | 1 |
| Down-regulated in J1 vs. J2 | GO:0005344 | oxygen carrier activity | 0.001108728 | 3 |
|  | GO:0019825 | oxygen binding | 0.001108728 | 3 |
|  | GO:0015020 | glucuronosyltransferase activity | 0.001108728 | 3 |
|  | GO:0015671 | oxygen transport | 0.001108728 | 3 |
|  | GO:0043231 | intracellular membrane-bounded organelle | 0.007008332 | 3 |
| Up-regulated in J2 vs. J3 | GO:0003700 | DNA-binding transcription factor activity | 0.002608526 | 23 |
|  | GO:0006355 | regulation of DNA-templated transcription | 0.002608526 | 34 |
|  | GO:0005667 | transcription regulator complex | 0.015155233 | 26 |
| Down-regulated in J2 vs. J3 | GO:0006508 | proteolysis | 0.008138891 | 13 |
|  | GO:0005506 | iron ion binding | 0.018143094 | 6 |
|  | GO:0016491 | oxidoreductase activity | 0.035963967 | 6 |
|  | GO:0020037 | heme binding | 0.040975881 | 6 |
|  | GO:0004252 | serine-type endopeptidase activity | 0.040975881 | 6 |
|  | GO:0008168 | methyltransferase activity | 0.040975881 | 4 |
|  | GO:0032259 | methylation | 0.047866659 | 4 |
| Up-regulated in J3 vs. A | GO:0007187 | G protein-coupled receptor signaling pathway, coupled to cyclic nucleotide second messenger | 9.90513E-24 | 52 |
|  | GO:0004930 | G protein-coupled receptor activity | 6.35585E-19 | 48 |
|  | GO:0007186 | G protein-coupled receptor signaling pathway | 2.92411E-16 | 45 |
|  | GO:0034220 | ion transmembrane transport | 1.35938E-10 | 24 |
|  | GO:0060079 | excitatory postsynaptic potential | 2.32976E-10 | 22 |
|  | GO:0005887 | integral component of plasma membrane | 2.07818E-09 | 50 |
|  | GO:0007218 | neuropeptide signaling pathway | 5.82155E-09 | 21 |
|  | GO:0005249 | voltage-gated potassium channel activity | 1.05584E-08 | 13 |
|  | GO:0004888 | transmembrane signaling receptor activity | 1.05584E-08 | 17 |
|  | GO:0005886 | plasma membrane | 1.26833E-08 | 51 |
|  | GO:0004983 | neuropeptide Y receptor activity | 1.76157E-08 | 12 |
|  | GO:0008076 | voltage-gated potassium channel complex | 2.84583E-08 | 14 |
|  | GO:0071805 | potassium ion transmembrane transport | 4.40642E-08 | 19 |
|  | GO:0045211 | postsynaptic membrane | 6.21768E-08 | 13 |
|  | GO:0030054 | cell junction | 1.50244E-07 | 13 |
|  | GO:0007268 | chemical synaptic transmission | 2.83934E-07 | 28 |
|  | GO:0034765 | regulation of ion transmembrane transport | 8.88529E-07 | 11 |
|  | GO:0022848 | acetylcholine-gated cation-selective channel activity | 1.374E-06 | 9 |
|  | GO:0005234 | extracellularly glutamate-gated ion channel activity | 9.23746E-06 | 14 |
|  | GO:0005230 | extracellular ligand-gated ion channel activity | 9.76029E-06 | 11 |
|  | GO:0007166 | cell surface receptor signaling pathway | 0.000106061 | 17 |
|  | GO:0007165 | signal transduction | 0.000543302 | 38 |
|  | GO:0035235 | ionotropic glutamate receptor signaling pathway | 0.00128487 | 19 |
|  | GO:0005918 | septate junction | 0.001347839 | 8 |
|  | GO:0098794 | postsynapse | 0.001707509 | 10 |
|  | GO:0004970 | ionotropic glutamate receptor activity | 0.002080192 | 20 |
|  | GO:0035556 | intracellular signal transduction | 0.002187895 | 27 |
|  | GO:0019991 | septate junction assembly | 0.00325591 | 8 |
|  | GO:0045503 | dynein light chain binding | 0.004837285 | 8 |
|  | GO:0004114 | 3',5'-cyclic-nucleotide phosphodiesterase activity | 0.004837285 | 6 |
|  | GO:0043565 | sequence-specific DNA binding | 0.004837285 | 33 |
|  | GO:0018108 | peptidyl-tyrosine phosphorylation | 0.005256618 | 11 |
|  | GO:0016020 | membrane | 0.005943244 | 64 |
|  | GO:0043005 | neuron projection | 0.007443982 | 7 |
|  | GO:0051260 | protein homooligomerization | 0.007651203 | 6 |
|  | GO:0005272 | sodium channel activity | 0.009118651 | 9 |
|  | GO:0006811 | ion transport | 0.009371639 | 21 |
|  | GO:0098793 | presynapse | 0.010358428 | 7 |
|  | GO:0005509 | calcium ion binding | 0.011046981 | 35 |
|  | GO:0070588 | calcium ion transmembrane transport | 0.015622532 | 8 |
|  | GO:0004713 | protein tyrosine kinase activity | 0.018985019 | 5 |
|  | GO:0030425 | dendrite | 0.027179133 | 7 |
|  | GO:0005622 | intracellular anatomical structure | 0.027622309 | 33 |
|  | GO:0043025 | neuronal cell body | 0.03287327 | 8 |
|  | GO:0003341 | cilium movement | 0.034691756 | 6 |
|  | GO:0035725 | sodium ion transmembrane transport | 0.035189085 | 9 |
|  | GO:0030276 | clathrin binding | 0.043071051 | 5 |
| Down-regulated in J3 vs. A | GO:0003735 | structural constituent of ribosome | 4.40006E-08 | 38 |
|  | GO:0042254 | ribosome biogenesis | 1.88111E-07 | 33 |
|  | GO:0005763 | mitochondrial small ribosomal subunit | 1.17723E-06 | 11 |
|  | GO:0032543 | mitochondrial translation | 1.44207E-05 | 12 |
|  | GO:0005762 | mitochondrial large ribosomal subunit | 1.94659E-05 | 11 |
|  | GO:0008137 | NADH dehydrogenase (ubiquinone) activity | 0.00452817 | 7 |
|  | GO:0006123 | mitochondrial electron transport, cytochrome c to oxygen | 0.00452817 | 6 |
|  | GO:0006120 | mitochondrial electron transport, NADH to ubiquinone | 0.005648708 | 8 |
|  | GO:0006412 | translation | 0.005648708 | 20 |
|  | GO:0043161 | proteasome-mediated ubiquitin-dependent protein catabolic process | 0.008316137 | 18 |
|  | GO:0046933 | proton-transporting ATP synthase activity, rotational mechanism | 0.010494411 | 6 |
|  | GO:0031625 | ubiquitin protein ligase binding | 0.01342617 | 16 |
|  | GO:0005743 | mitochondrial inner membrane | 0.016444736 | 14 |
|  | GO:0030162 | regulation of proteolysis | 0.018962235 | 12 |
|  | GO:0004298 | threonine-type endopeptidase activity | 0.021096359 | 6 |
|  | GO:0004252 | serine-type endopeptidase activity | 0.03456047 | 22 |
|  | GO:0008289 | lipid binding | 0.03456047 | 7 |
|  | GO:0019005 | SCF ubiquitin ligase complex | 0.03456047 | 12 |
|  | GO:0006744 | ubiquinone biosynthetic process | 0.03456047 | 7 |
|  | GO:0015986 | proton motive force-driven ATP synthesis | 0.049669201 | 6 |

Table S3. Contingency table for comparisons of embryo-specific highly expressed genes (HEGs) and genes not belonging to embryo-specific HEGs for genes in the “lightcyan” module and not in the “lightcyan” module. A set of 12,061 genes with the sum of DESeq2-normalized read counts of at least 10 was included in this analysis.

| Gene set | Embryo-specific HEGs | Genes not belonging to embryo-specific HEGs | Total |
| --- | --- | --- | --- |
| Genes in “lightcyan” module | 99 | 555 | 654 |
| Genes not in “lightcyan” module | 165 | 11,242 | 11,407 |
| Total | 264 | 11,797 | 12,061 |

Table S4. List of hub genes at the embryonic stage. These genes were ranked by degree of connectivity (from high to low). Gene ID, name, annotation source (from UniProt or NR databases) and full name of each gene is provided. Genes associated with cell proliferation are shown in bold.

| Gene ID | Name | Source | Full name |
| --- | --- | --- | --- |
| D_mitsukuri10400 | *Uncharacterized* | NR | hypothetical protein DAPPUDRAFT_302188 |
| D_mitsukuri07039 | *HMMR* | NR | hyaluronan-mediated motility receptor-like isoform X2 |
| **D_mitsukuri07840** | ***INCENP-B*** | **UniProt** | **Inner centromere protein B** |
| **D_mitsukuri07392** | ***RBBP4-B*** | **UniProt** | **Histone-binding protein RBBP4-B** |
| **D_mitsukuri07313** | ***KIF14*** | **UniProt** | **Kinesin-like protein KIF14** |
| D_mitsukuri01645 | *LAM* | UniProt | Lamin Dm0 |
| D_mitsukuri12345 | *MAP1LC3C* | UniProt | Microtubule-associated proteins 1A/1B light chain 3C |
| **D_mitsukuri07600** | ***NET1*** | **UniProt** | **Neuroepithelial cell-transforming gene 1 protein** |
| D_mitsukuri05723 | *Uncharacterized* | NR | hypothetical protein DAPPUDRAFT_229040 |
| **D_mitsukuri11159** | ***MCM4*** | **UniProt** | **DNA replication licensing factor MCM4** |
| **D_mitsukuri05046** | ***UHRF2*** | **UniProt** | **E3 ubiquitin-protein ligase UHRF2** |
| D_mitsukuri10895 | *ARHGAP11A* | UniProt | Rho GTPase-activating protein 11A |
| D_mitsukuri08743 | *LBR* | UniProt | Delta(14)-sterol reductase LBR |
| D_mitsukuri01225 | *TOP2B* | UniProt | DNA topoisomerase 2-beta |
| **D_mitsukuri09797** | ***MCM7*** | **UniProt** | **DNA replication licensing factor MCM7** |
| **D_mitsukuri04611** | ***MCM2*** | **UniProt** | **DNA replication licensing factor MCM2** |
| **D_mitsukuri03641** | ***AURKA-B*** | **UniProt** | **Aurora kinase A-B** |
| **D_mitsukuri04666** | ***ANLN*** | **UniProt** | **Anillin** |
| D_mitsukuri09614 | *ALYREF2* | UniProt | Aly/REF export factor 2 |
| **D_mitsukuri07633** | ***KIF23*** | **UniProt** | **Kinesin-like protein KIF23** |

Table S5. GO enrichment terms of genes in gene-expression patterns P2-P5 at the juvenile developmental stages.

| Expression pattern | GO id | GO terms | FDR-corrected *P*-value | Number of genes |
| --- | --- | --- | --- | --- |
| P2 | GO:0008020 | G protein-coupled photoreceptor activity | 8.34245E-07 | 8 |
|  | GO:0007187 | G protein-coupled receptor signaling pathway, coupled to cyclic nucleotide second messenger | 8.34245E-07 | 16 |
|  | GO:0009584 | detection of visible light | 1.85092E-06 | 7 |
|  | GO:0007602 | phototransduction | 2.19451E-05 | 6 |
|  | GO:0005887 | integral component of plasma membrane | 4.36076E-05 | 18 |
|  | GO:0034765 | regulation of ion transmembrane transport | 6.84951E-05 | 6 |
|  | GO:0007601 | visual perception | 9.06175E-05 | 6 |
|  | GO:0005230 | extracellular ligand-gated ion channel activity | 0.000169024 | 6 |
|  | GO:0008076 | voltage-gated potassium channel complex | 0.000291223 | 6 |
|  | GO:0034220 | ion transmembrane transport | 0.000481576 | 8 |
|  | GO:0005249 | voltage-gated potassium channel activity | 0.001167771 | 5 |
|  | GO:0004930 | G protein-coupled receptor activity | 0.001396459 | 11 |
|  | GO:0070588 | calcium ion transmembrane transport | 0.004790424 | 5 |
|  | GO:0007186 | G protein-coupled receptor signaling pathway | 0.006140053 | 10 |
|  | GO:0035556 | intracellular signal transduction | 0.006386218 | 11 |
|  | GO:0071805 | potassium ion transmembrane transport | 0.006959595 | 6 |
|  | GO:0005622 | intracellular anatomical structure | 0.008308579 | 14 |
|  | GO:0005509 | calcium ion binding | 0.009154345 | 14 |
|  | GO:0004888 | transmembrane signaling receptor activity | 0.010420535 | 5 |
|  | GO:0045202 | synapse | 0.01250569 | 4 |
|  | GO:0050804 | modulation of chemical synaptic transmission | 0.02122862 | 3 |
|  | GO:0030286 | dynein complex | 0.026246075 | 3 |
|  | GO:0048488 | synaptic vesicle endocytosis | 0.026246075 | 3 |
|  | GO:0051260 | protein homooligomerization | 0.032954417 | 3 |
|  | GO:0007218 | neuropeptide signaling pathway | 0.043654416 | 5 |
|  | GO:0006887 | exocytosis | 0.048582498 | 3 |
| P3 | GO:0006525 | arginine metabolic process | 0.001047602 | 4 |
|  | GO:0006560 | proline metabolic process | 0.016672339 | 3 |
| P4 | GO:0016788 | hydrolase activity, acting on ester bonds | 0.02220566 | 3 |
|  | GO:0016042 | lipid catabolic process | 0.02220566 | 3 |
|  | GO:0005794 | Golgi apparatus | 0.034077769 | 4 |
| p5 | GO:0004252 | serine-type endopeptidase activity | 0.000232079 | 10 |
|  | GO:0006508 | proteolysis | 0.000804008 | 15 |
|  | GO:0016788 | hydrolase activity, acting on ester bonds | 0.025053386 | 3 |
|  | GO:0033041 | sweet taste receptor activity | 0.025053386 | 3 |
|  | GO:0004984 | olfactory receptor activity | 0.025053386 | 3 |
|  | GO:0030425 | dendrite | 0.025053386 | 3 |
|  | GO:0016042 | lipid catabolic process | 0.025053386 | 3 |
|  | GO:0007635 | chemosensory behavior | 0.025053386 | 3 |
|  | GO:0010037 | response to carbon dioxide | 0.025053386 | 3 |
|  | GO:0050913 | sensory perception of bitter taste | 0.025053386 | 3 |
|  | GO:0008049 | male courtship behavior | 0.025053386 | 3 |
|  | GO:0030424 | axon | 0.030698351 | 3 |
|  | GO:0006629 | lipid metabolic process | 0.038569462 | 4 |
|  | GO:0098794 | postsynapse | 0.038706327 | 3 |
|  | GO:0043025 | neuronal cell body | 0.040663565 | 3 |
|  | GO:0005794 | Golgi apparatus | 0.042041779 | 4 |

Table S6. Contingency table for comparisons of genes in P1 and not in P1 for genes in the “magenta” module and not in the “magenta” module. A set of 12,061 genes with the sum of DESeq2-normalized read counts of at least 10 was included in this analysis.

| Gene set | Genes in P1 | Genes not in P1 | Total |
| --- | --- | --- | --- |
| Genes in “magenta” module | 522 | 925 | 1,447 |
| Genes not in “magenta” module | 566 | 10,048 | 10,614 |
| Total | 1,088 | 10,973 | 12,061 |

Table S7. List of hub genes at the juvenile developmental stages. These genes were ranked by degree of connectivity (from high to low). Gene ID, name, annotation source (from UniProt or NR databases) and full name of each gene is provided. Genes associated with neurotransmission are shown in bold.

| Gene ID | Name | Source | Full name |
| --- | --- | --- | --- |
| D_mitsukuri01168 | *SORBS1* | UniProt | Sorbin and SH3 domain-containing protein 1 |
| **D_mitsukuri01923** | ***ZIG-8*** | **UniProt** | **Zwei Ig domain protein zig-8** |
| **D_mitsukuri07999** | ***FMRFAMIDE*** | **NR** | **FMRFamide perprohormone** |
| D_mitsukuri01625 | *BAG* | NR | BAG family molecular chaperone regulator |
| **D_mitsukuri09499** | ***HTR1*** | UniProt | **5-hydroxytryptamine receptor 1** |
| D_mitsukuri05249 | *PLEKHG7* | UniProt | Pleckstrin homology domain-containing family G member 7 |
| D_mitsukuri12724 | *PER* | UniProt | Period circadian protein |
| D_mitsukuri12245 | *Uncharacterized* | NR | Uncharacterized protein APZ42_011519 |
| D_mitsukuri04206 | *GMII* | UniProt | Alpha-mannosidase 2 |
| **D_mitsukuri05493** | ***SYT1*** | **UniProt** | **Synaptotagmin-1** |
| D_mitsukuri01782 | *CACNA2D4* | UniProt | Voltage-dependent calcium channel subunit alpha-2/delta-4 |
| D_mitsukuri01430 | *SPR* | UniProt | Sex peptide receptor |
| **D_mitsukuri01079** | ***SOL1*** | **UniProt** | **Suppressor of lurcher protein 1** |
| D_mitsukuri02140 | *REM1* | UniProt | GTP-binding protein REM 1 |
| D_mitsukuri01696 | *NPR1* | UniProt | Atrial natriuretic peptide receptor 1 |
| **D_mitsukuri00375** | ***NACHRALPHA2*** | **UniProt** | **Acetylcholine receptor subunit alpha-like 2** |
| D_mitsukuri01268 | *CADM3* | UniProt | Cell adhesion molecule 3 |
| D_mitsukuri08257 | *SIX1B* | UniProt | Homeobox protein six1b |
| **D_mitsukuri00371** | ***DSCAM2*** | **UniProt** | **Down syndrome cell adhesion molecule-like protein Dscam2** |
| D_mitsukuri12715 | *IQCA1* | UniProt | Dynein regulatory complex protein 11 |

Table S8. Contingency table for comparisons of adult-specific highly expressed genes (HEGs) and genes not belonging to adult-specific HEGs for genes in the “darkolivegreen” module and not in the “darkolivegreen” module. A set of 12,061 genes with the sum of DESeq2-normalized read counts of at least 10 was included in this analysis.

| Gene set | Adult-specific HEGs | Genes not belonging to adult-specific HEGs | Total |
| --- | --- | --- | --- |
| Genes in “darkolivegreen” module | 177 | 267 | 444 |
| Genes not in “darkolivegreen” module | 473 | 11,144 | 11,617 |
| Total | 650 | 11,411 | 12,061 |

Table S9. List of hub genes at the adult developmental stage. These genes were ranked by degree of connectivity (from high to low). Gene ID, name, annotation source (from UniProt or NR databases) and full name of each gene is provided. Genes associated with activities against oxidative stress are shown in bold.

| Gene ID | Name | Source | Full name |
| --- | --- | --- | --- |
| D_mitsukuri06664 | *Uncharacterized* | NR | Hypothetical protein DAPPUDRAFT_266377 |
| D_mitsukuri01125 | *TMPRSS9* | UniProt | Transmembrane protease serine 9 |
| D_mitsukuri12662 | *VG* | UniProt | Vitellogenin |
| **D_mitsukuri10241** | ***QDPR*** | **UniProt** | **Dihydropteridine reductase** |
| D_mitsukuri01176 | *SUN* | UniProt | Protein stunted |
| D_mitsukuri11822 | *ACBD7* | UniProt | Acyl-CoA-binding domain-containing protein 7 |
| D_mitsukuri06446 | *NDUFB4* | NR | NADH dehydrogenase [ubiquinone] 1 beta subcomplex subunit 4 |
| D_mitsukuri05809 | *COMMD7* | UniProt | COMM domain-containing protein 7 |
| D_mitsukuri12648 | *AIF1* | NR | Apoptosis-inducing factor 1 |
| D_mitsukuri00325 | *TMEM14C* | UniProt | Transmembrane protein 14C |
| **D_mitsukuri04178** | ***SOD1*** | **UniProt** | **Superoxide dismutase [Cu-Zn]** |
| D_mitsukuri07675 | *PEBP1* | UniProt | Phosphatidylethanolamine-binding protein 1 |
| D_mitsukuri00009 | *TMPRSS11D* | UniProt | Transmembrane protease serine 11D |
| D_mitsukuri11734 | *APT1* | UniProt | Adenine phosphoribosyltransferase |
| D_mitsukuri00100 | *CYOP* | UniProt | Probable cytosolic oligopeptidase A |
| D_mitsukuri13371 | *NPC2A* | UniProt | NPC intracellular cholesterol transporter 2 homolog A |
| D_mitsukuri06707 | *CTRP3* | NR | C1q and tumor necrosis factor-related protein-like protein 3 |
| D_mitsukuri06971 | *Uncharacterized* | NR | Hypothetical protein DAPPUDRAFT_306131 |
| **D_mitsukuri07832** | ***GSTM1*** | **UniProt** | **Glutathione S-transferase Mu 1** |
| D_mitsukuri12773 | *Uncharacterized* | NR | Hypothetical protein DAPPUDRAFT_303708 |

Table S10. Contingency table for comparisons of highly expressed genes (HEGs; or P1 genes) and non-HEGs (or non-P1 genes) at the embryonic stage, at the juvenile stages and at the adult stage for genes in expanded versus non-expanded gene families. A set of 12,670 genes with datable expression (at least one sample with DESeq2-normalized read counts ≥ 1) in the development of *Daphnia mitsukuri* was included in this analysis.

|  |  | Genes in expanded families | Genes in non-expanded families | Total |
| --- | --- | --- | --- | --- |
| Embryonic stage | HEGs | 4 | 260 | 264 |
|  | non-HEGs | 478 | 11,928 | 12,406 |
|  | Total | 482 | 12,188 | 12,670 |
| Juvenile stage | P1 genes | 54 | 1,034 | 1,088 |
|  | non-P1 genes | 428 | 11,154 | 11,582 |
|  | Total | 482 | 12,188 | 12,670 |
| Adult stage | HEGs | 19 | 425 | 444 |
|  | non-HEGs | 463 | 11,763 | 12,226 |
|  | Total | 482 | 12,188 | 12,670 |

Table S11. List of 54 P1 genes at the juvenile developmental stages that also belong to expanded gene families of *Daphnia* species. Gene ID, name, annotation source (from UniProt or NR databases) and full name of each gene is provided. Genes encoding rhodopsin are shown in bold.

| Gene ID | Name | Source | Full name |
| --- | --- | --- | --- |
| D_mitsukuri00157 | *PCE* | UniProt | Proclotting enzyme |
| D_mitsukuri00227 | *STAR* | UniProt | Protein Star |
| D_mitsukuri00229 | *TOPORS* | UniProt | E3 ubiquitin-protein ligase Topors |
| D_mitsukuri00267 | *TIM* | UniProt | Protein timeless |
| **D_mitsukuri00276** | ***RHO*** | **UniProt** | **Rhodopsin** |
| D_mitsukuri00760 | *ZSWIM3* | UniProt | Zinc finger SWIM domain-containing protein 3 |
| D_mitsukuri00995 | *ABCG23* | UniProt | ABC transporter G family member 23 |
| D_mitsukuri01003 | *Uncharacterized* | NR | EFX70378.1 hypothetical protein DAPPUDRAFT_257192 |
| D_mitsukuri01200 | *BRACHYURIN* | UniProt | Brachyurin |
| D_mitsukuri01232 | *FLT1* | UniProt | Vascular endothelial growth factor receptor 1 |
| D_mitsukuri01334 | *FLT4* | UniProt | Vascular endothelial growth factor receptor 3 |
| D_mitsukuri01454 | *Uncharacterized* | NR | KZS17527.1 Uncharacterized protein APZ42_016400 |
| D_mitsukuri01516 | *Uncharacterized* | NR | EFX72921.1 hypothetical protein DAPPUDRAFT_253739 |
| D_mitsukuri01531 | *FLT4* | UniProt | Vascular endothelial growth factor receptor 3 |
| D_mitsukuri02428 | *GAL3ST1* | UniProt | Galactosylceramide sulfotransferase |
| D_mitsukuri02756 | *GRID2* | UniProt | Glutamate receptor ionotropic, delta-2 |
| D_mitsukuri02771 | *CG5065* | UniProt | Putative fatty acyl-CoA reductase CG5065 |
| D_mitsukuri02778 | *ABCG20* | UniProt | ABC transporter G family member 20 |
| D_mitsukuri02854 | *GR* | UniProt | Glutamate receptor |
| D_mitsukuri03001 | *Uncharacterized* | NR | KZS10317.1 Uncharacterized protein APZ42_025234 |
| D_mitsukuri03207 | *Uncharacterized* | NR | EFX74057.1 hypothetical protein DAPPUDRAFT_252358 |
| D_mitsukuri03211 | *Uncharacterized* | NR | KZS14302.1 Uncharacterized protein APZ42_020377 |
| D_mitsukuri03306 | *CASD1* | UniProt | N-acetylneuraminate 9-O-acetyltransferase |
| D_mitsukuri03474 | *UGT2A2* | UniProt | UDP-glucuronosyltransferase 2A2 |
| D_mitsukuri04067 | *Uncharacterized* | NR | EFX80331.1 hypothetical protein DAPPUDRAFT_303990 |
| **D_mitsukuri04087** | ***RHO*** | **UniProt** | **Rhodopsin** |
| **D_mitsukuri04088** | ***RHO*** | **UniProt** | **Rhodopsin** |
| **D_mitsukuri04090** | ***RHO*** | **UniProt** | **Rhodopsin** |
| **D_mitsukuri04091** | ***RHO*** | **UniProt** | **Rhodopsin** |
| D_mitsukuri04228 | *Uncharacterized* | NR | EFX80481.1 hypothetical protein DAPPUDRAFT_304150 |
| D_mitsukuri04230 | *Uncharacterized* | NR | KZS21247.1 Uncharacterized protein APZ42_011855 |
| **D_mitsukuri05134** | ***RHO*** | **UniProt** | **Rhodopsin** |
| D_mitsukuri05526 | *Uncharacterized* | NR | EFX89819.1 hypothetical protein DAPPUDRAFT_310129 |
| D_mitsukuri06288 | *GRIK3* | UniProt | Glutamate receptor ionotropic, kainate 3 |
| D_mitsukuri06947 | *B3GNT6* | UniProt | Acetylgalactosaminyl-O-glycosyl-glycoprotein beta-1,3-N-acetylglucosaminyltransferase |
| D_mitsukuri06954 | *FUCTC* | UniProt | Alpha-(1,3)-fucosyltransferase C |
| D_mitsukuri07990 | *VMO1* | UniProt | Vitelline membrane outer layer protein 1 homolog |
| D_mitsukuri09360 | *Uncharacterized* | NR | EFX84319.1 hypothetical protein DAPPUDRAFT_239062 |
| **D_mitsukuri09428** | ***RHO*** | **UniProt** | **Rhodopsin** |
| D_mitsukuri09678 | *HTR1F* | UniProt | 5-hydroxytryptamine receptor 1F |
| **D_mitsukuri09711** | ***RHO*** | **UniProt** | **Rhodopsin** |
| **D_mitsukuri09715** | ***RHO*** | **UniProt** | **Rhodopsin** |
| D_mitsukuri10267 | *NT1* | UniProt | Neurotrophin 1 |
| D_mitsukuri10269 | *NT1* | UniProt | Neurotrophin 1 |
| D_mitsukuri10486 | *Uncharacterized* | NR | KZS14282.1 Uncharacterized protein APZ42_020464 |
| D_mitsukuri11065 | *Uncharacterized* | NR | KZS19986.1 Uncharacterized protein APZ42_013442 |
| D_mitsukuri12244 | *TC1A* | UniProt | Transposable element Tc1 transposase |
| D_mitsukuri12530 | *Uncharacterized* | NR | EFX79766.1 hypothetical protein DAPPUDRAFT_319198 |
| D_mitsukuri12862 | *TOPORS* | UniProt | E3 ubiquitin-protein ligase Topors |
| D_mitsukuri13520 | *CUE* | UniProt | Protein cueball |
| D_mitsukuri13522 | *DPEP1* | UniProt | Dipeptidase 1 |
| D_mitsukuri13531 | *CUE* | UniProt | Protein cueball |
| D_mitsukuri13532 | *CUE* | UniProt | Protein cueball |
| D_mitsukuri13729 | *TOPORS* | UniProt | E3 ubiquitin-protein ligase Topors |

Table S12. Contingency table for comparisons of highly expressed genes (HEGs; or P1 genes) and non-HEGs (or non-P1 genes) at the embryonic stage, at the juvenile stages and at the adult stage for genes in *Daphnia*-specific versus not in *Dphnia*-specific gene families. A set of 12,670 genes with datable expression (at least one sample with DESeq2-normalized read counts ≥ 1) in the development of *Daphnia mitsukuri* was included in this analysis.

|  |  | Genes in *Daphnia*-specific gene families | Genes not in *Daphnia*-specific gene families | Total |
| --- | --- | --- | --- | --- |
| Embryonic stage | HEGs | 22 | 242 | 264 |
|  | non-HEGs | 857 | 11,549 | 12,406 |
|  | Total | 879 | 11,791 | 12,670 |
| Juvenile stage | P1 genes | 64 | 1,024 | 1,088 |
|  | non-P1 genes | 815 | 10,767 | 11,582 |
|  | Total | 879 | 11,791 | 12,670 |
| Adult stage | HEGs | 50 | 394 | 444 |
|  | non-HEGs | 829 | 11,397 | 12,226 |
|  | Total | 879 | 11,791 | 12,670 |

Table S13. List of 50 highly expressed genes at the adult developmental stage that also belong to specific gene families in *Daphnia* species. Gene ID, name, annotation source (from UniProt or NR databases) and full name of each gene is provided.

| Gene ID | Name | Source | Full name |
| --- | --- | --- | --- |
| D_mitsukuri00030 | *Uncharacterized* | NR | EFX78310.1 hypothetical protein DAPPUDRAFT_105377 |
| D_mitsukuri00033 | *Uncharacterized* | NR | EFX78310.1 hypothetical protein DAPPUDRAFT_105377 |
| D_mitsukuri00571 | *Uncharacterized* | NR | EFX70093.1 hypothetical protein DAPPUDRAFT_300588 |
| D_mitsukuri01108 | *Uncharacterized* | NR | EFX79521.1 hypothetical protein DAPPUDRAFT_304477 |
| D_mitsukuri01126 | *PRP1* | UniProt | Repetitive proline-rich cell wall protein 1 |
| D_mitsukuri01127 | *PRP1* | UniProt | Repetitive proline-rich cell wall protein 1 |
| D_mitsukuri01131 | *ZBTB26* | UniProt | Zinc finger and BTB domain-containing protein 26 |
| D_mitsukuri01175 | *Uncharacterized* | NR | EFX79578.1 hypothetical protein DAPPUDRAFT_104306 |
| D_mitsukuri01186 | *Uncharacterized* | NR | EFX79578.1 hypothetical protein DAPPUDRAFT_104306 |
| D_mitsukuri01442 | *Uncharacterized* | NR | EFX82564.1 hypothetical protein DAPPUDRAFT_101160 |
| D_mitsukuri01455 | *Uncharacterized* | NR | KZS18681.1 Uncharacterized protein APZ42_015232 |
| D_mitsukuri01884 | *Uncharacterized* | NR | EFX89955.1 hypothetical protein DAPPUDRAFT_309625 |
| D_mitsukuri04166 | *Uncharacterized* | NR | EFX80517.1 hypothetical protein DAPPUDRAFT_304031 |
| D_mitsukuri04179 | *BRN* | UniProt | Beta-1,3-galactosyltransferase brn |
| D_mitsukuri05937 | *Uncharacterized* | NR | EFX74193.1 hypothetical protein DAPPUDRAFT_307462 |
| D_mitsukuri05944 | *Uncharacterized* | NR | EFX74189.1 hypothetical protein DAPPUDRAFT_324590 |
| D_mitsukuri06477 | *TC1A* | UniProt | Transposable element Tc1 transposase |
| D_mitsukuri06817 | *Uncharacterized* | NR | EFX82133.1 hypothetical protein DAPPUDRAFT_241234 |
| D_mitsukuri06842 | *Uncharacterized* | NR | EFX82114.1 hypothetical protein DAPPUDRAFT_302599 |
| D_mitsukuri06971 | *Uncharacterized* | NR | EFX76519.1 hypothetical protein DAPPUDRAFT_306131 |
| D_mitsukuri07015 | *Uncharacterized* | NR | EFX76448.1 hypothetical protein DAPPUDRAFT_306181 |
| D_mitsukuri07136 | *Uncharacterized* | NR | EFX68622.1 hypothetical protein DAPPUDRAFT_301273 |
| D_mitsukuri07404 | *Uncharacterized* | NR | EFX76155.1 hypothetical protein DAPPUDRAFT_226158 |
| D_mitsukuri07756 | *Uncharacterized* | NR | EFX88317.1 hypothetical protein DAPPUDRAFT_305505 |
| D_mitsukuri08019 | *CREBBP* | UniProt | CREB-binding protein |
| D_mitsukuri08315 | *VGP* | NR | EFX65042.1 vertebrate gliacolin-like protein |
| D_mitsukuri08551 | *Uncharacterized* | NR | EFX84485.1 hypothetical protein DAPPUDRAFT_99654 |
| D_mitsukuri09013 | *Uncharacterized* | None | Uncharacterized protein |
| D_mitsukuri09073 | *Uncharacterized* | NR | EFX81692.1 hypothetical protein DAPPUDRAFT_317375 |
| D_mitsukuri09232 | *Uncharacterized* | NR | EFX81629.1 hypothetical protein DAPPUDRAFT_241929 |
| D_mitsukuri09308 | *GLBD* | UniProt | Globin D, coelomic |
| D_mitsukuri09380 | *Uncharacterized* | NR | EFX83956.1 hypothetical protein DAPPUDRAFT_314997 |
| D_mitsukuri09427 | *Uncharacterized* | NR | EFX83990.1 hypothetical protein DAPPUDRAFT_315059 |
| D_mitsukuri09603 | *GLBD* | UniProt | Globin D, coelomic |
| D_mitsukuri09618 | *Uncharacterized* | NR | EFX83704.1 hypothetical protein DAPPUDRAFT_301601 |
| D_mitsukuri09656 | *Uncharacterized* | NR | EFX83786.1 hypothetical protein DAPPUDRAFT_301647 |
| D_mitsukuri09929 | *TC1A* | UniProt | Transposable element Tc1 transposase |
| D_mitsukuri10363 | *Uncharacterized* | NR | EFX67137.1 hypothetical protein DAPPUDRAFT_302196 |
| D_mitsukuri10365 | *Uncharacterized* | NR | EFX67137.1 hypothetical protein DAPPUDRAFT_302196 |
| D_mitsukuri10366 | *Uncharacterized* | NR | EFX67129.1 hypothetical protein DAPPUDRAFT_331396 |
| D_mitsukuri10382 | *Uncharacterized* | NR | EFX67137.1 hypothetical protein DAPPUDRAFT_302196 |
| D_mitsukuri10603 | *LUCI* | UniProt | Luciferin 4-monooxygenase |
| D_mitsukuri11097 | *Uncharacterized* | NR | EFX79213.1 hypothetical protein DAPPUDRAFT_304912 |
| D_mitsukuri11422 | *BICC1* | UniProt | Protein bicaudal C homolog 1 |
| D_mitsukuri11428 | *Uncharacterized* | NR | EFX75454.1 hypothetical protein DAPPUDRAFT_107952 |
| D_mitsukuri11631 | *ELOF* | UniProt | Elongation of very long chain fatty acids protein |
| D_mitsukuri11959 | *Uncharacterized* | NR | EFX89165.1 hypothetical protein DAPPUDRAFT_310683 |
| D_mitsukuri12011 | *Uncharacterized* | NR | EFX89141.1 hypothetical protein DAPPUDRAFT_310727 |
| D_mitsukuri12057 | *Uncharacterized* | NR | EFX89099.1 hypothetical protein DAPPUDRAFT_220751 |
| D_mitsukuri12435 | *Uncharacterized* | NR | EFX66322.1 hypothetical protein DAPPUDRAFT_263368 |

Table S14. Statistics of annotated protein-coding genes in the *Daphnia mitsukuri* genome.

| Public Database | Number of annotated genes | Percentage of annotated genes relative to all predicted genes (%) |
| --- | --- | --- |
| NCBI NR database | 13,121 | 91.7 |
| UniProt | 9,133 | 63.8 |
| KOG | 9,399 | 65.7 |
| KEGG | 5,284 | 36.9 |
| Pfam | 9,345 | 66.6 |
| Gene ontology annotation | 9,466 | 66.2 |
| Total | 13,179 | 93.9 |

Figure S1. WGCNA hierarchical clustering dendrogram of genes (with DESeq2-normalized read count at least 10) in the *Daphnia mitsukuri* genome. Y-axis represents the co-expression distance between genes while x-axis represents individual genes. Twenty distinct co-expression modules were identified and denoted by colored bars at the bottom of the dendrogram.


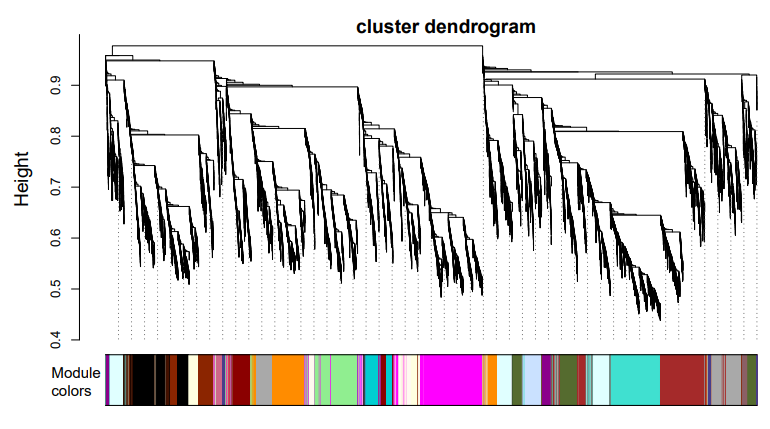


Figure S2. Correlations between module eigengenes and developmental stages in *D. mitsukuri* according to the WGCNA analysis. Boxes are colored by strength of correlation. The number of genes residing in each module is given in parentheses. The numbers within the heat map represent correlations and FDR-corrected *P*-values (in parentheses; red, positively correlated, blue, negatively correlated) for the module-period associations.


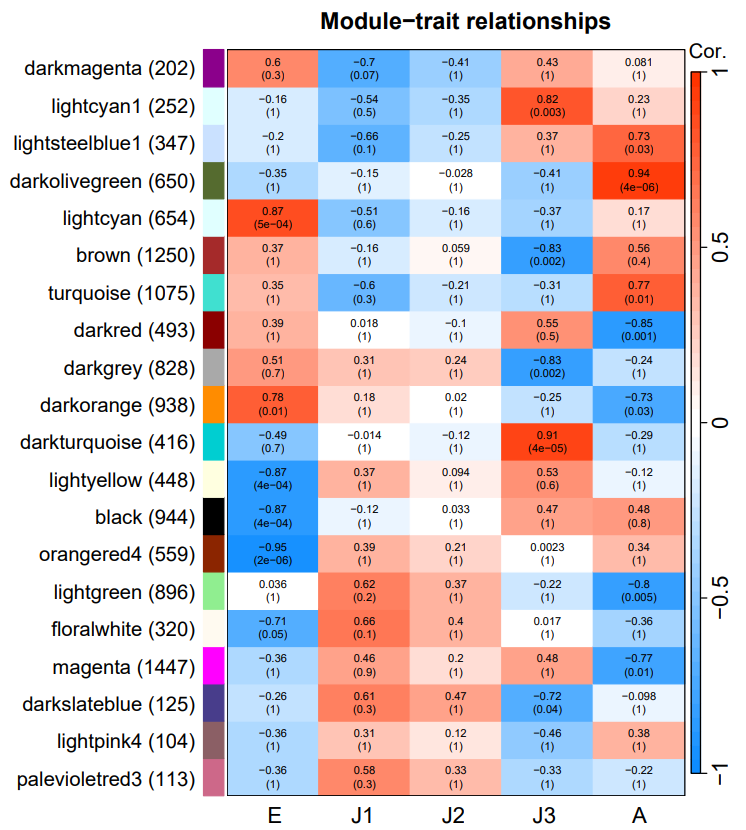


Figure S3. GO enrichment terms of the 264 embryo-specific highly expressed genes in the development of *Daphnia mitsukuri*. Number of genes in each GO category is shown in the center of the circle.


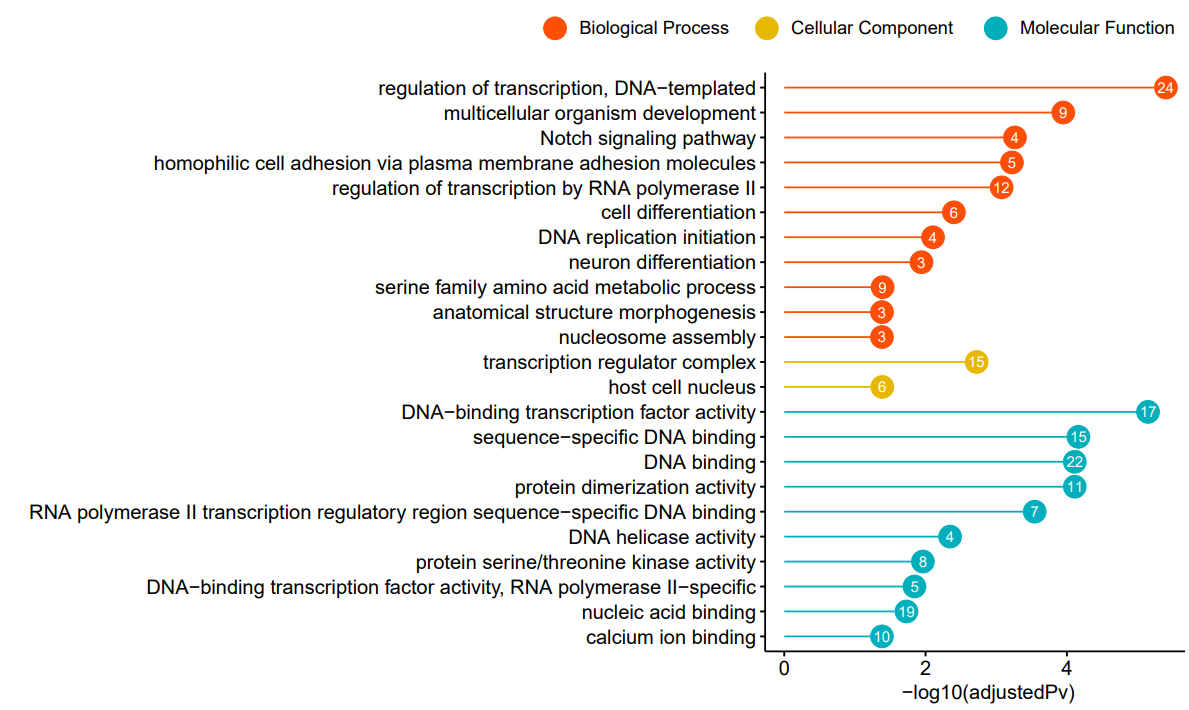


Figure S4. Enriched KEGG terms for the 264 embryo-specific highly expressed genes in the development of *Daphnia mitsukuri*. The number of genes in each KEGG pathway is shown on the right of the bar. KEGG pathways associated with cell proliferation or cell differentiation are shown in bold.


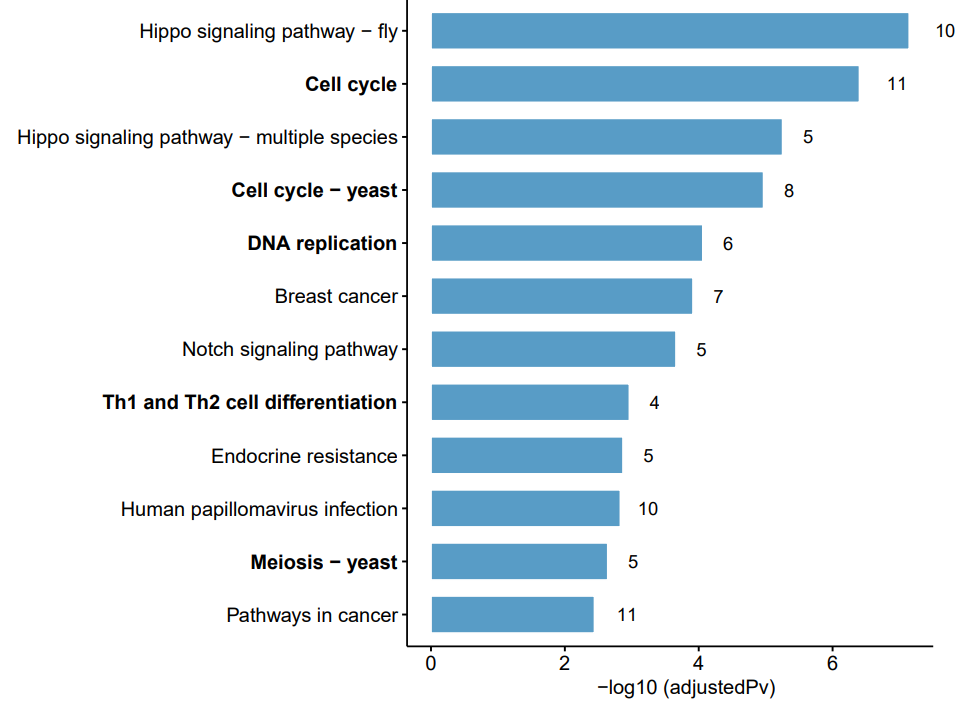


Figure S5. GO enrichment terms of embryo-specific highly expressed genes as well as being in the “lightcyan” module. Number of genes in each GO category is shown in the center of the circle.


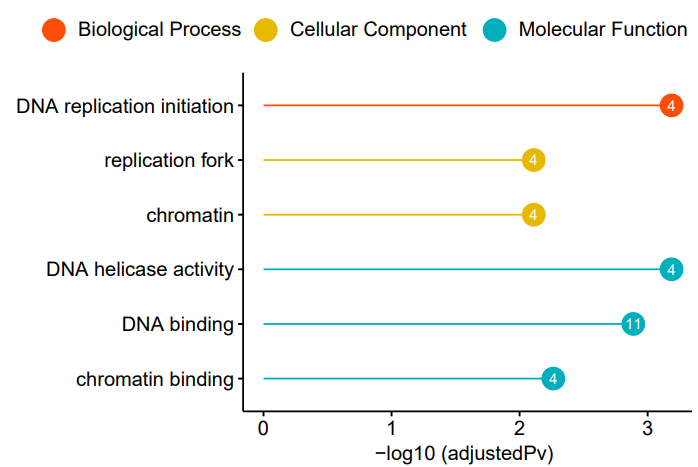


Figure S6. GO enrichment terms of genes that belong to the “lightcyan” module but are not highly expressed at the embryonic stage. Number of genes in each GO category is shown in the center of the circle.


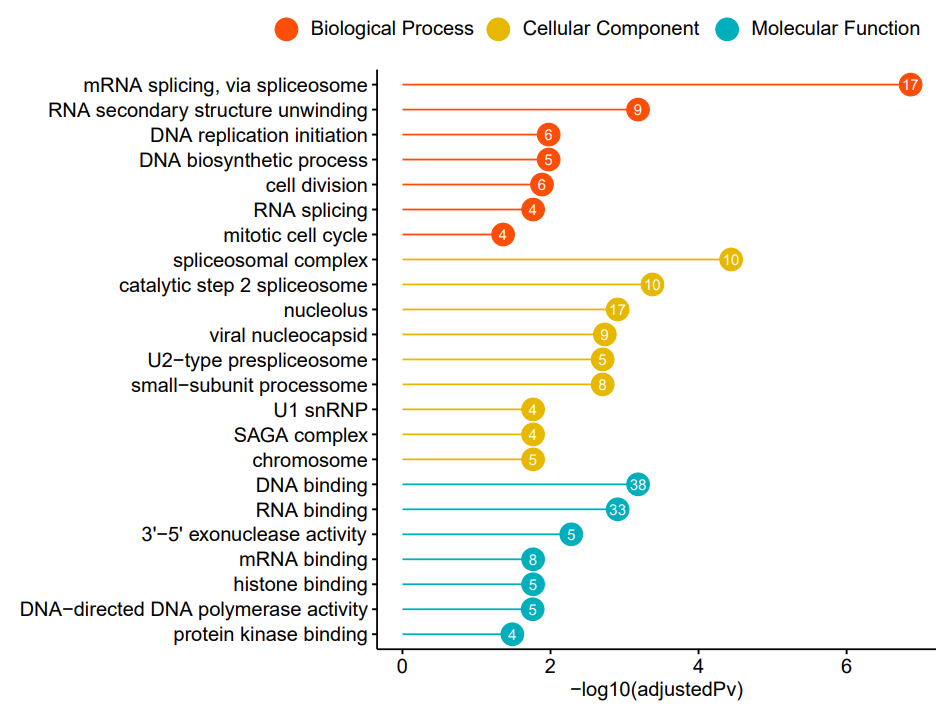


Figure S7. GO enrichment terms of highly expressed genes at the embryonic stage that did not belong to the “lightcyan” module. Number of genes in each GO category is shown in the center of the circle.


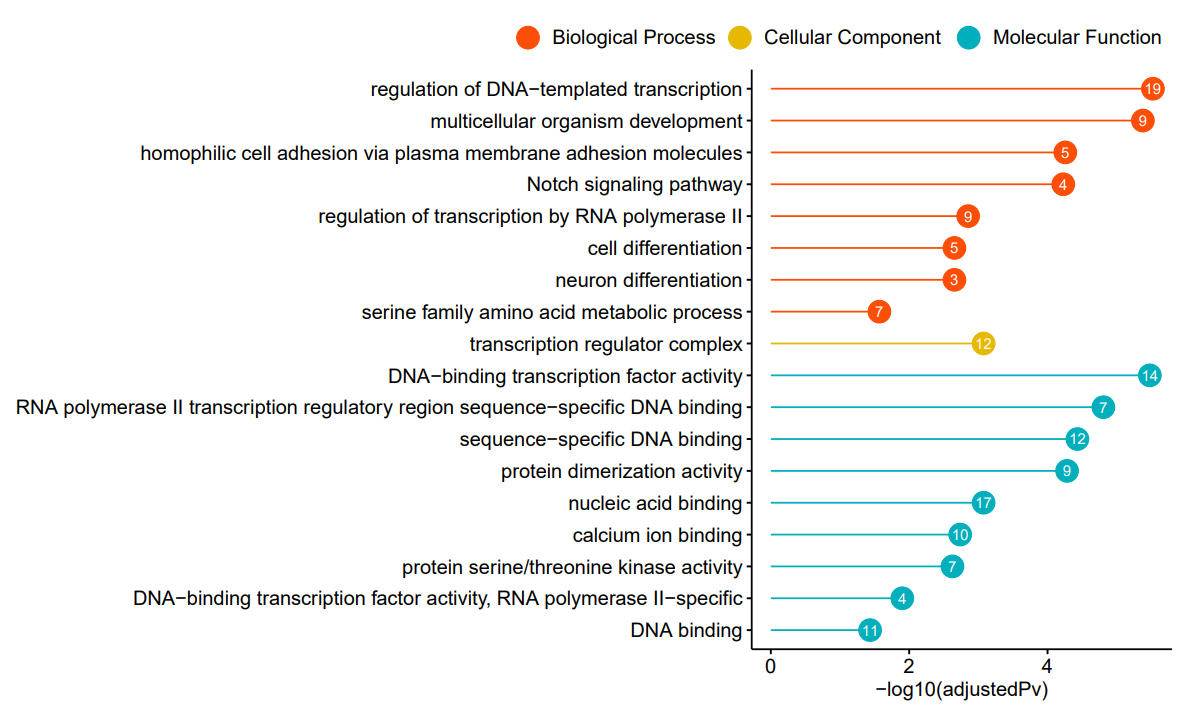


Figure S8. GO enrichment terms of genes in P1 class (high expression across J1, J2 and J3) at the juvenile developmental stages. Number of genes in each GO category is shown in the center of the circle.


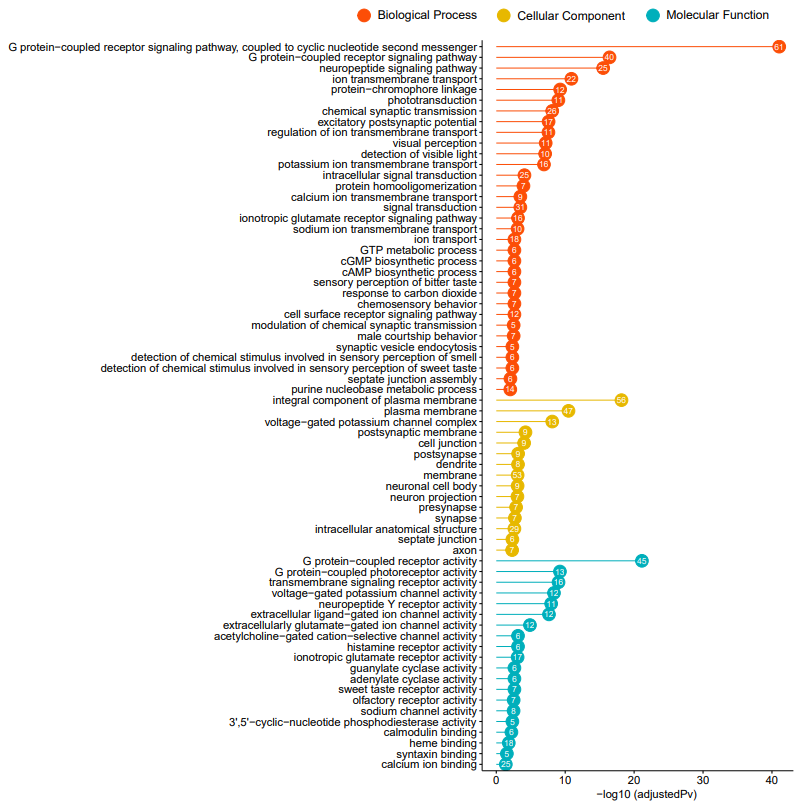


Figure S9. Enriched KEGG terms of genes in P1 class (high expression across J1, J2 and J3). The number of genes in each KEGG pathway is shown on the right of the bar.


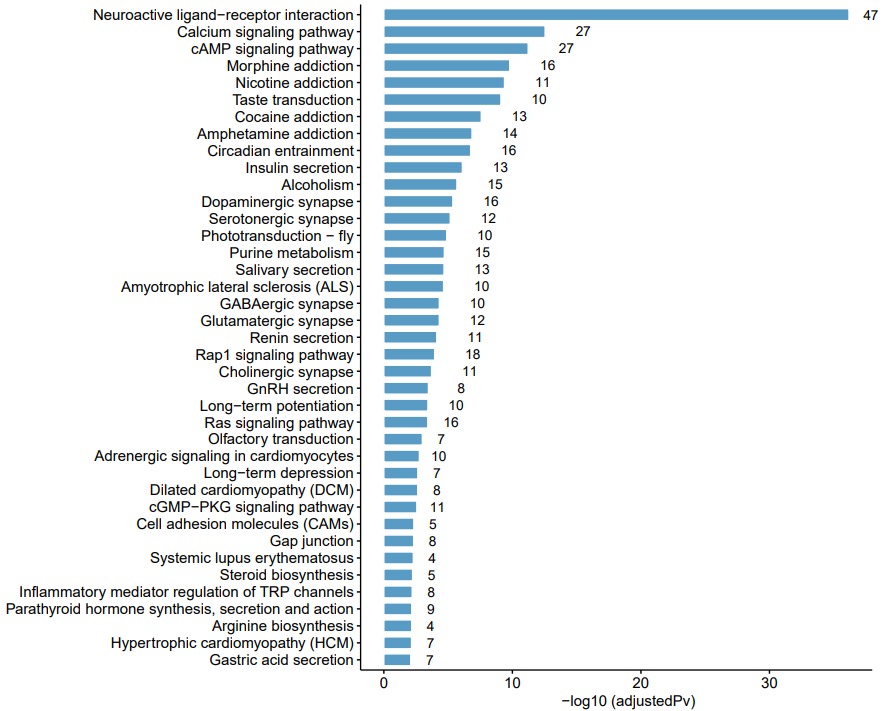


Figure S10. GO enrichment terms of genes that belong to P1 class as well as to the “magenta” module. Number of genes in each GO category is shown in the center of the circle.


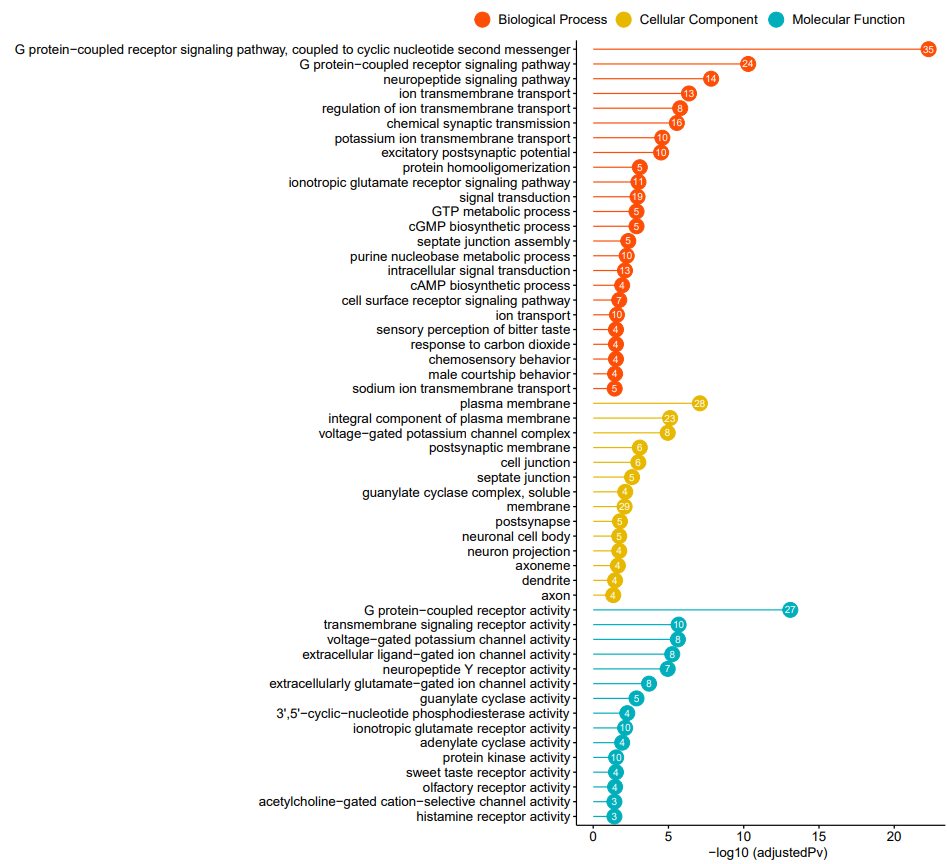


Figure S11. GO enrichment terms of genes that belong to the “magenta” module but are not P1 genes at the juvenile developmental stages. Number of genes in each GO category is shown in the center of the circle.


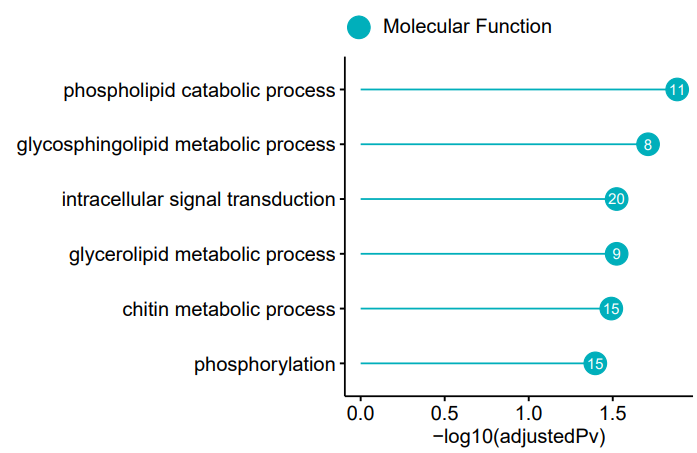


Figure S12. GO enrichment terms of P1 genes that did not belong to the “magenta” module at the juvenile developmental stages. Number of genes in each GO category is shown in the center of the circle.


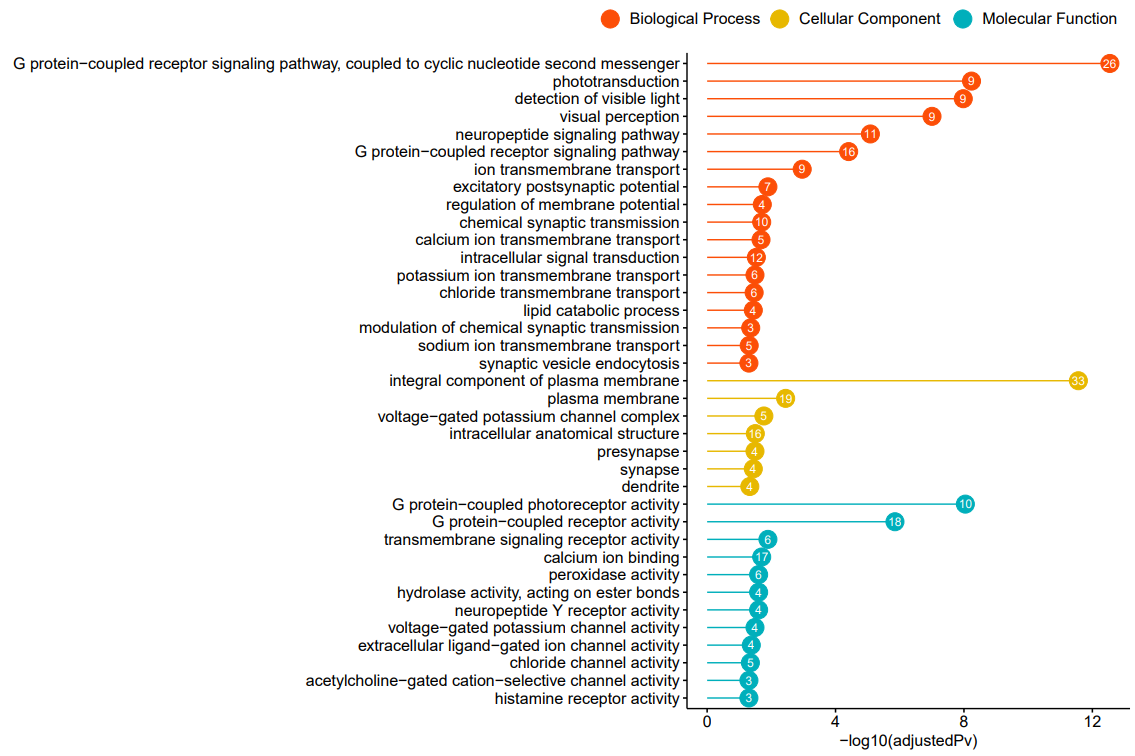


Figure S13. GO enrichment terms of adult-specific highly expressed genes in the development of *Daphnia mitsukuri*. Number of genes in each GO category is shown in the center of the circle.


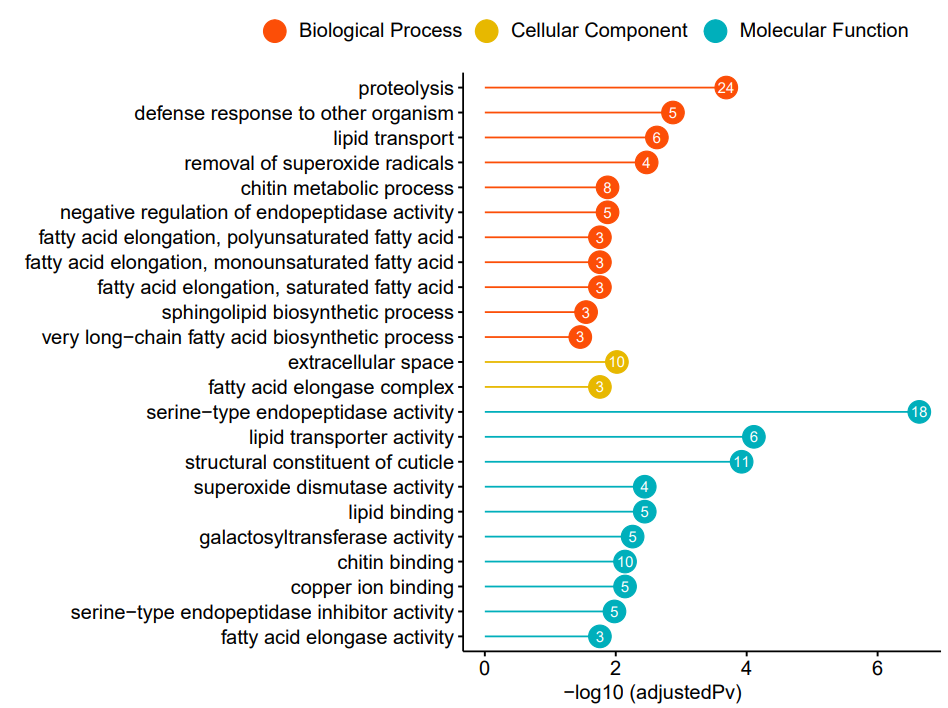


Figure S14. GO enrichment terms of genes that belong to adult-specific highly expressed genes as well as to the “darkolivegreen” module. Number of genes in each GO category is shown in the center of the circle.


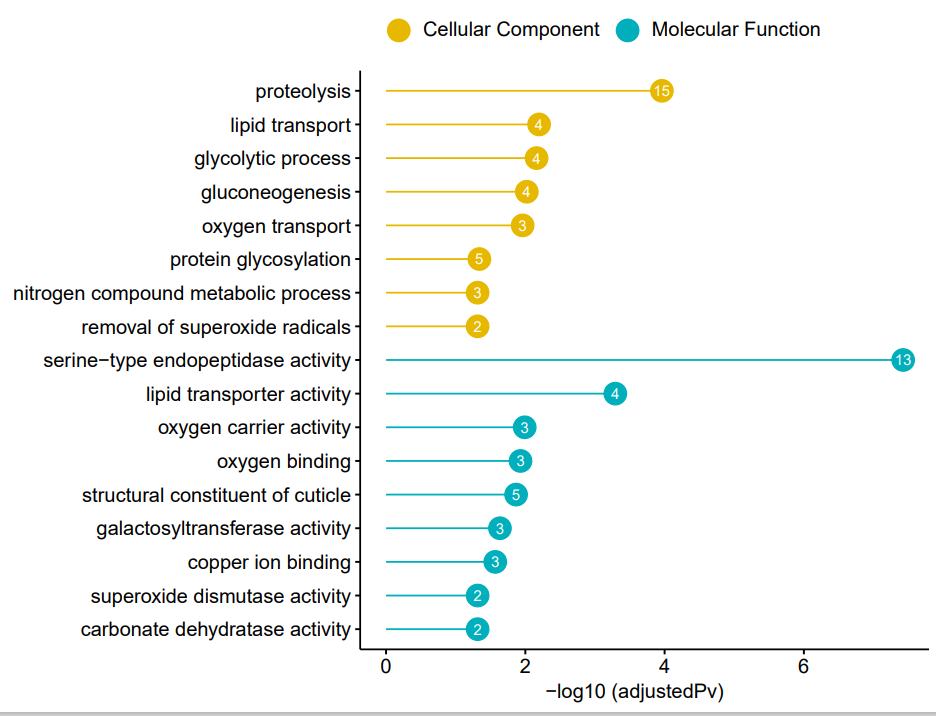


Figure S15. GO enrichment terms of genes that belong to the “darkolivegreen” module but are not HEGs at the adult developmental stage. Number of genes in each GO category is shown in the center of the circle.


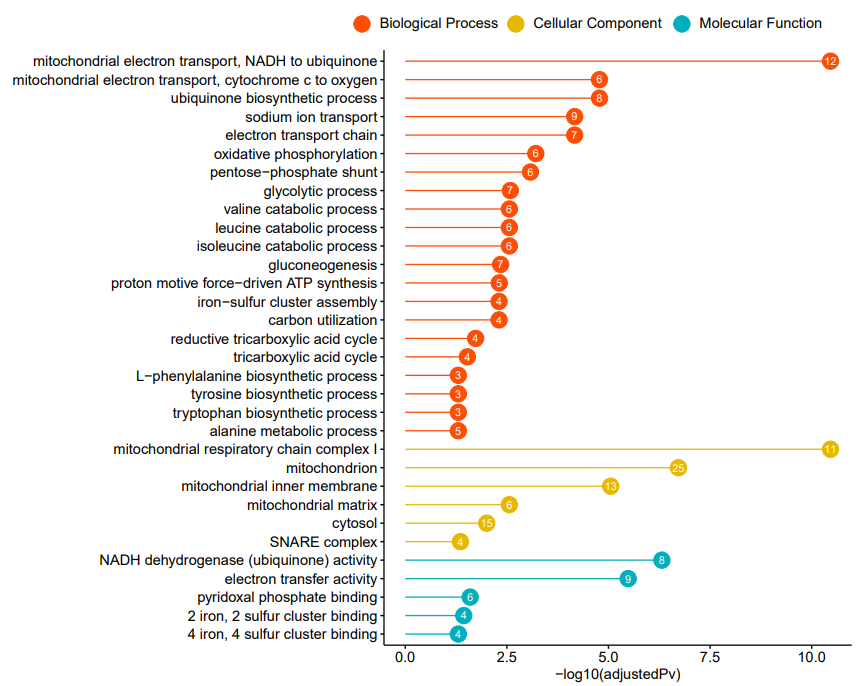


Figure S16. GO enrichment terms of highly expressed genes at the adult stage that did not belong to the “darkolivegreen” module. Number of genes in each GO category is shown in the center of the circle.


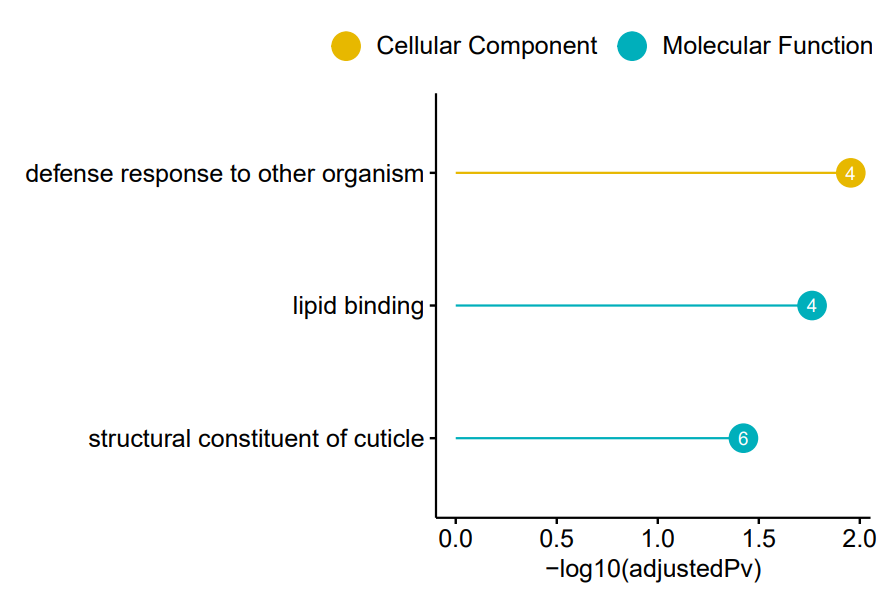


Figure S17. GO enrichment terms of the P1 genes at the juvenile developmental stages that also belong to expanded gene families of *Daphnia* species. Number of genes in each GO category is shown in the center of the circle.


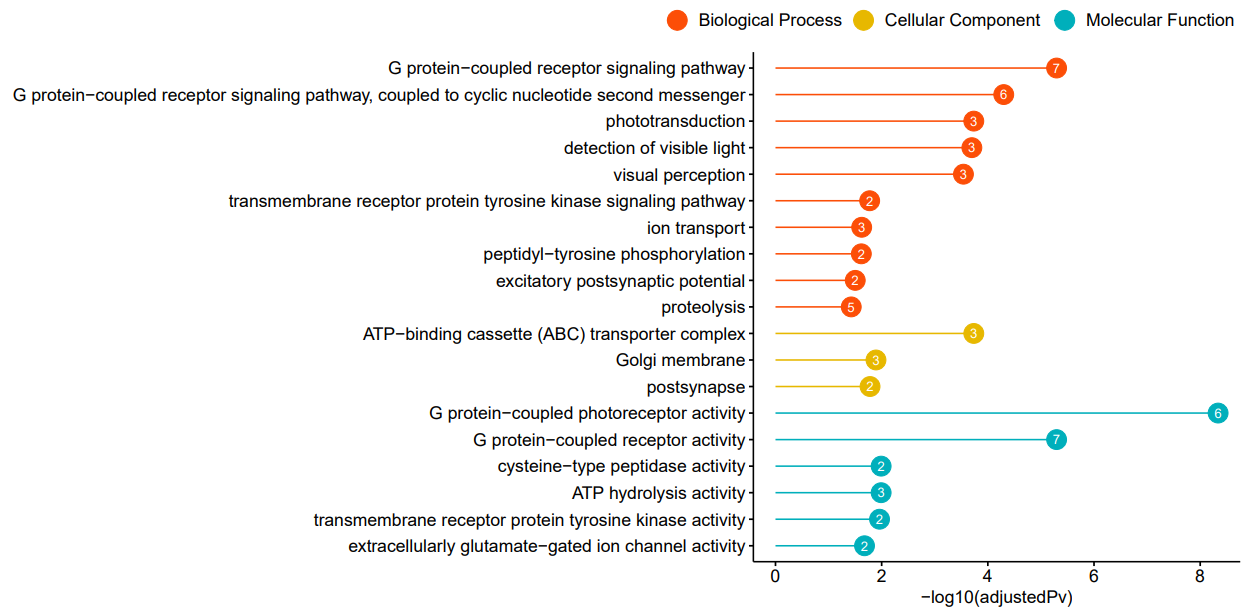


Figure S18. GO enrichment terms of the top 500 genes that drive the separation in the PCA analysis.


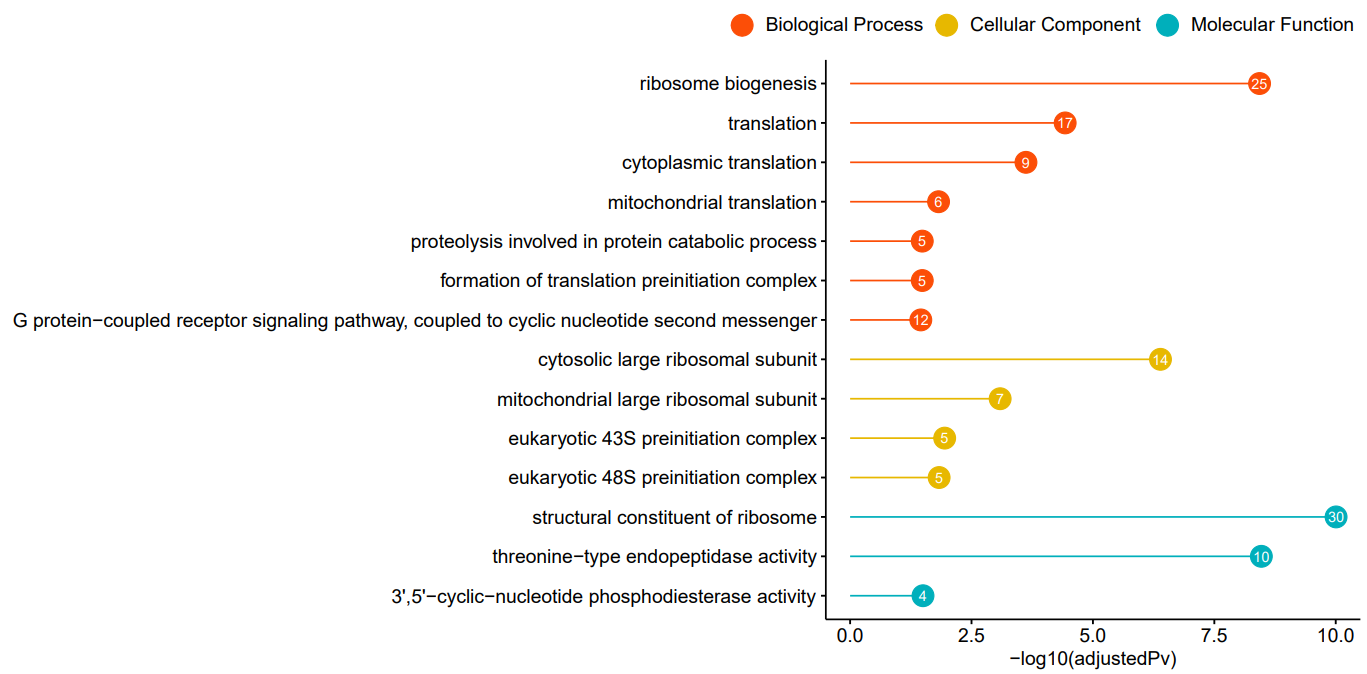


Figure S19. Gene-expression patterns underpinning the development of *Daphnia mitsukuri*. Fifty GO terms, among those enriched for HEGs or WGCNA modules and exhibiting a strong positive correlation with each developmental stage, were classified into five groups by function: DNA replication, differentiation, sensory perception, cuticle formation, stress responses. The expression levels of all the genes annotated to these GO terms in the genome are included. The vertical axes indicate log2 DEseq2 normalized counts. The horizontal axes indicate the stages. Error bars indicate confidence intervals.


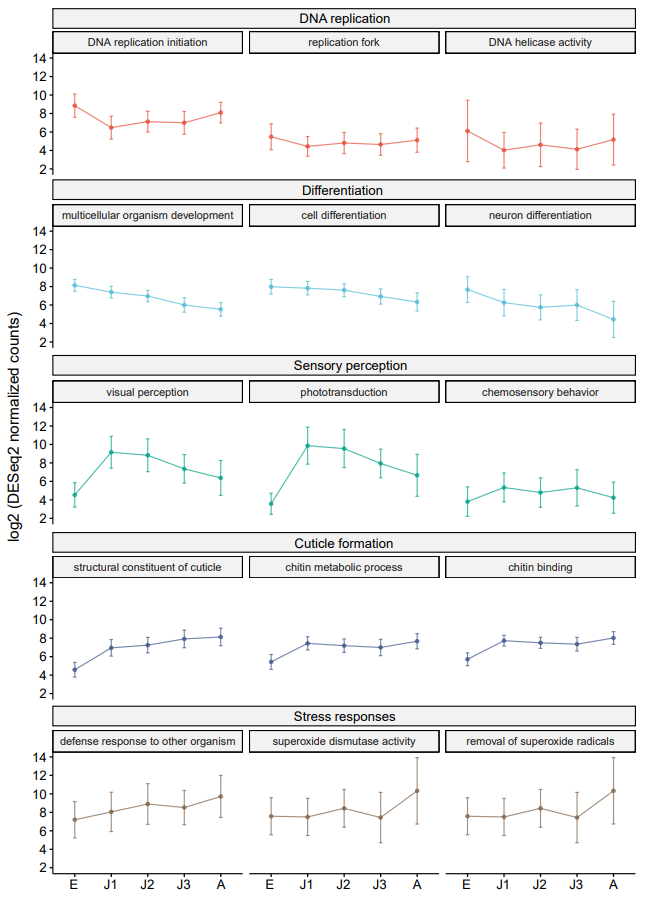

Supplement: Supplementary file 1 — Supplementary Material 1. [file 12864_2024_10210_MOESM1_ESM.docx]
